# Comparison of different group-level templates in gradient-based multimodal connectivity analysis

**DOI:** 10.1162/netn_a_00382

**Published:** 2024-12-10

**Authors:** Sunghun Kim, Seulki Yoo, Ke Xie, Jessica Royer, Sara Larivière, Kyoungseob Byeon, Jong Eun Lee, Yeongjun Park, Sofie L. Valk, Boris C. Bernhardt, Seok-Jun Hong, Hyunjin Park, Bo-yong Park

**Affiliations:** Department of Artificial Intelligence, Sungkyunkwan University, Suwon, Republic of Korea; Center for Neuroscience Imaging Research, Institute for Basic Science, Suwon, Republic of Korea; GE HealthCare Korea, Seoul, Republic of Korea; McConnell Brain Imaging Centre, Montreal Neurological Institute and Hospital, McGill University, Montreal, Quebec, Canada; Brigham and Women’s Hospital, Harvard Medical School, Boston, MA, USA; Center for the Integrative Developmental Neuroscience, Child Mind Institute, New York, NY, USA; Department of Electrical and Computer Engineering, Sungkyunkwan University, Suwon, Republic of Korea; Forschungszentrum Jülich, Jülich, Germany; Max Planck Institute for Cognitive and Brain Sciences, Leipzig, Germany; Department of Biomedical Engineering, Sungkyunkwan University, Suwon, Republic of Korea; School of Electronic and Electrical Engineering, Sungkyunkwan University, Suwon, Republic of Korea; Department of Brain and Cognitive Engineering, Korea University, Seoul, Republic of Korea

**Keywords:** Connectome gradients, Gradient alignment, Group-level template, Multimodal connectivity analysis

## Abstract

The study of large-scale brain connectivity is increasingly adopting unsupervised approaches that derive low-dimensional spatial representations from high-dimensional connectomes, referred to as gradient analysis. When translating this approach to study interindividual variations in connectivity, one technical issue pertains to the selection of an appropriate group-level template to which individual gradients are aligned. Here, we compared different group-level template construction strategies using functional and structural connectome data from neurotypical controls and individuals with autism spectrum disorder (ASD) to identify between-group differences. We studied multimodal magnetic resonance imaging data obtained from the Autism Brain Imaging Data Exchange (ABIDE) Initiative II and the Human Connectome Project (HCP). We designed six template construction strategies that varied in whether (1) they included typical controls in addition to ASD; or (2) they mapped from one dataset onto another. We found that aligning a combined subject template of the ASD and control subjects from the ABIDE Initiative onto the HCP template exhibited the most pronounced effect size. This strategy showed robust identification of ASD-related brain regions for both functional and structural gradients across different study settings. Replicating the findings on focal epilepsy demonstrated the generalizability of our approach. Our findings will contribute to improving gradient-based connectivity research.

## Author Summary

Gradient-based connectivity analysis provides a compact understanding of complex connectivity patterns across the brain. One issue of the gradient analysis is to choose an appropriate group-level template in which individual gradients are aligned. Here, we assessed six different strategies for constructing group-level gradient templates, including those based on combined data from individuals with psychiatric conditions and control subjects, and data from an independent dataset of young, healthy adults. The choice of template significantly influences the outcomes of gradient analyses, with templates combining data from both groups or aligning to a high-quality independent dataset, providing more balanced results. This study emphasizes the importance of template selection in brain connectivity studies, contributing to more reliable gradient analyses.

## INTRODUCTION

Recent progress in analytical techniques for multimodal magnetic resonance imaging (MRI) has encouraged the investigation of whole-brain structural and functional organization in vivo. Brain connectivity analysis is a representative method for understanding the interregional relationships of brain structure and function (Bullmore & Sporns, 2009; Rubinov & Sporns, 2010; Smith et al., 2011). Functional connectivity evaluates the cofluctuations of brain signals measured by functional MRI by calculating the correlations of the time series between different brain regions (Biswal et al., 1995; Bullmore & Sporns, 2009; Fox & Raichle, 2007; Sporns et al., 2005). On the other hand, structural connectivity based on diffusion MRI tractography estimates the neuronal fiber tracts connecting different regions (Bullmore & Sporns, 2009; Sporns et al., 2005).

A recent study applied a nonlinear dimensionality reduction method to analyze complex connectome data (Margulies et al., 2016). The key idea of this approach is to project a high-dimensional connectivity matrix onto a low-dimensional manifold space by generating multiple low-dimensional eigenvectors, often referred to as “gradients” (Bernhardt et al., 2022; Haak & Beckmann, 2020; Haak et al., 2018; Huntenburg et al., 2018; Margulies et al., 2016). The estimated gradients may provide overlapping connectome organization patterns along the whole brain and depict continual changes in connectivity. Gradients estimated from functional connectivity exhibit smooth transitions along the cortical surface, showing multiple axes of sensory-transmodal, visual-somatomotor, and multiple-demand networks in the rest of the brain (Margulies et al., 2016; Vos de Wael et al., 2020). Additionally, gradients of the structural connectome show medial-lateral, inferior–superior, and anterior–posterior axes of the brain, and the findings have been related to different aspects of the functional organization (Park, Bethlehem, et al., 2021; Park, Hong, et al., 2021; Park, Vos de Wael, et al., 2021).

Gradient-based connectivity analysis has been increasingly used to examine large-scale brain reorganization in multiple neurological and psychiatric conditions, including autism spectrum disorder (ASD), schizophrenia, and epilepsy (Hong et al., 2019; Park, Hong, et al., 2021; Tian et al., 2019). The gradient values of individuals in diseased and healthy cohorts are often compared to investigate between-group differences in the continuously changing connectivity patterns along the cortical surface. To allow comparison of individual-level gradients in group-level analysis, it is necessary to align the gradients of individuals to a common template gradient (Langs et al., 2015; Nenning et al., 2020; Vos de Wael et al., 2020; Xu et al., 2020). However, despite prior studies on calibrating gradient generation for biomarker discovery (Hong et al., 2020), there is currently no consensus regarding which template to use. One possible strategy is to compute a group-level template based on averaged functional or structural connectivity across all subjects or group-representative structural connectivity derived from a distance-dependent thresholding approach (Betzel et al., 2019), to which both diagnostic groups are aligned. Another method is to align both healthy and diseased subjects onto a template that is defined using only healthy populations. However, which strategy is better remains debated, and quantitative evaluations are largely absent.

This study compared different group-level template gradient construction strategies using multimodal connectome data of neurotypical controls and individuals with ASD. We constructed different templates using (1) both control subjects and individuals with ASD from the same database, (2) only control subjects, (3) only individuals with ASD, (4) young, healthy adults from an independent dataset with high image quality, (5) control and ASD aligned to the independent young, healthy adults, and (6) only controls aligned to the independent young, healthy adults. We aimed to determine a reasonable template for gradient analysis in contrasting groups and identify the template construction strategy that is most effective in identifying between-group differences and significant brain clusters. Additionally, to verify our findings, we conducted a replication study using multimodal connectome data from healthy controls and individuals with focal epilepsy.

## RESULTS

### Study Participants

We studied 59 individuals with ASD and 56 neurotypical controls obtained from the Autism Brain Imaging Data Exchange Initiative (ABIDE-II; https://fcon_1000.projects.nitrc.org/indi/abide; Di Martino et al., 2017). We also analyzed multimodal MRI data from the Human Connectome Project (HCP) database (Van Essen, Ugurbil, et al., 2012) as an additional control dataset. Demographic information is listed in Supplementary Table 1 in the Supporting Information. See the Methods section for detailed subject selection criteria.

### Functional Connectivity Gradients

**Figure 1.**
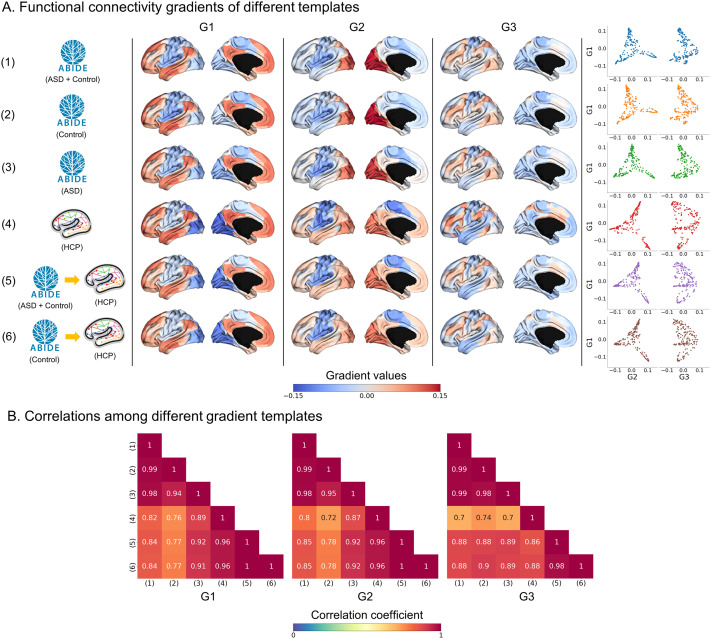
Functional connectivity gradients of different templates and their similarity. (A) We generated three functional connectivity gradients (G1, G2, and G3) of six different template strategies and plotted them on brain surfaces. The scatterplots show the distribution of gradient pairs. (B) The heatmap of correlation coefficients between different template pairs. The spin permutation tests showed that all pairs were significant at the significance level of 0.05. Abbreviations: ABIDE, Autism Brain Imaging Data Exchange Initiative; ASD, autism spectrum disorder; HCP, Human Connectome Project.

We constructed the functional connectivity matrix via linear correlation of time series between different brain regions and applied a nonlinear manifold learning technique to generate low-dimensional eigenvectors (i.e., gradients). The three functional gradients (G1, G2, and G3) accounted for approximately 47.3% of the information in the input connectivity matrix (Supplementary Table 2). G1 showed a gradual axis with sensory and motor systems on one end and transmodal regions, such as the default mode and frontoparietal networks, on the other. G2 showed a somatomotor-to-visual axis, and G3 differentiated the multiple-demand networks from the rest of the brain (Figure 1A). Overall, the spatial patterns of the gradients were similar across different templates (1,000 spin permutation tests, *p* < 0.05 for all cases; Figure 1B). The correlations between the templates (1) ASD + control (ABIDE) and (2) control (ABIDE), as well as between (5) ASD + control (ABIDE) → HCP and (6) control (ABIDE) → HCP, showed the highest similarity across the three gradients (mean ± *SD* *r* = 0.99 ± 0.01). In contrast, the lowest similarity was found between (2) control (ABIDE) and (4) HCP (*r* = 0.74 ± 0.02).

### Between-Group Differences in Functional Gradients

**Figure 2.**
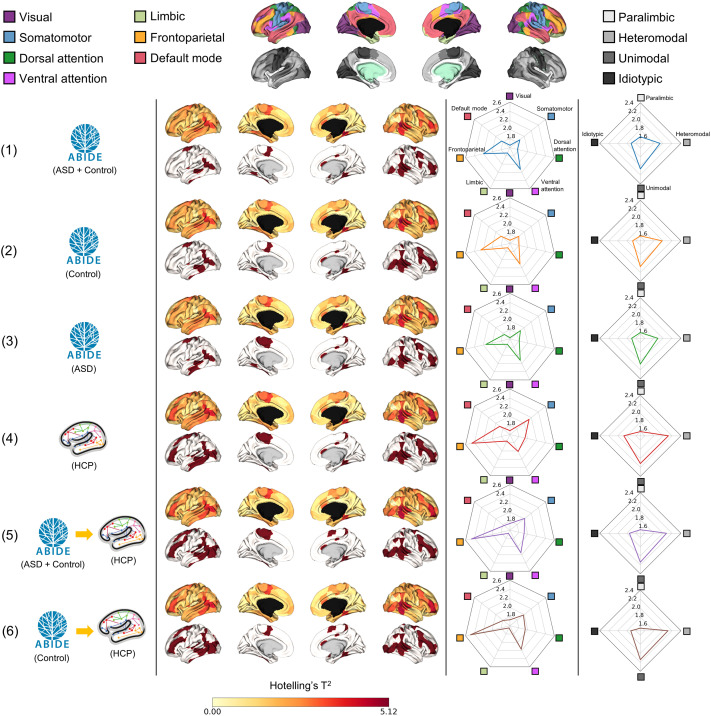
Between-group differences in the first three functional connectivity gradients between individuals with ASD and neurotypical controls based on different template gradients. Hotelling’s *T*^2^ statistics of the whole brain are plotted on brain surfaces. The regions showed significant (*p*_FDR_ < 0.05) between-group differences in three gradients marked in red. We stratified the effects according to seven functional communities and four cortical hierarchical levels using radar plots. Abbreviations: ABIDE, Autism Brain Imaging Data Exchange Initiative; ASD, autism spectrum disorder; HCP, Human Connectome Project.

We performed cortex-wide multivariate analysis to explore significant differences (*p*_FDR_ < 0.05) in the three functional gradients between individuals with ASD and neurotypical controls (Figure 2). Across different gradient templates, significant between-group differences were observed in the middle temporal cortex and sensory regions such as the visual and auditory areas. On the other hand, aligning data to the HCP template and its variants resulted in stronger effect sizes in terms of Hotelling’s *T*^2^ (mean ± *SD* statistics = 1.88 ± 0.85 for Templates 1, 2, 3 averaged; 1.98 ± 0.93 for Templates 4, 5, 6 averaged) and the number of significant regions (i.e., Template 1 = 36; Template 2 = 38; Template 3 = 39; Template 4 = 48; Template 5 = 54; Template 6 = 53). Indeed, the HCP template and its variants additionally showed between-group differences in the dorsolateral prefrontal cortex compared with the ABIDE templates. We stratified the between-group difference effects according to seven intrinsic functional communities (visual, somatomotor, dorsal attention, ventral attention, limbic, frontoparietal, and default mode) (Yeo et al., 2011) and four cortical hierarchical levels (idiotypic, unimodal, heteromodal association, and paralimbic) (Mesulam, 1998) (Figure 2). In general, the frontoparietal network had the strongest between-group differences (mean ± *SD* statistics = 2.34 ± 0.15; across templates), followed by the ventral attention (2.12 ± 0.05) and somatomotor (1.96 ± 0.11) networks. The unimodal (2.10 ± 0.04; across templates) and heteromodal association (2.02 ± 0.01) areas showed strong effects concerning the cortical hierarchical levels. Details on specific between-group differences within functional communities can be found in Supplementary Figure 1A. Overall, in the functional connectivity gradients, effect sizes obtained using the HCP template and its variants (mean ± *SD* statistics = 2.00 ± 0.93 [Template 4]; 1.97 ± 0.93 [Template 5]; 1.97 ± 0.94 [Template 6]; across 200 parcels) were larger than those observed using templates derived from the ABIDE dataset (1.89 ± 0.85 [Template 1]; 1.90 ± 0.86 [Template 2]; 1.87 ± 0.85 [Template 3]). However, when comparing the pure HCP Template 4 with its variants 5 and 6, reduced effects were observed in the default mode network (mean statistics = 1.89 [Template 4] → 1.76 [5], 1.79 [6]; 6.88%, 5.29% decrease) and somatomotor (2.14 [Template 4] → 2.01 [5], 1.98 [6]; 6.07%, 7.48% decrease) for the HCP variants.

### Structural Connectivity Gradients and Between-Group Differences

**Figure 3.**
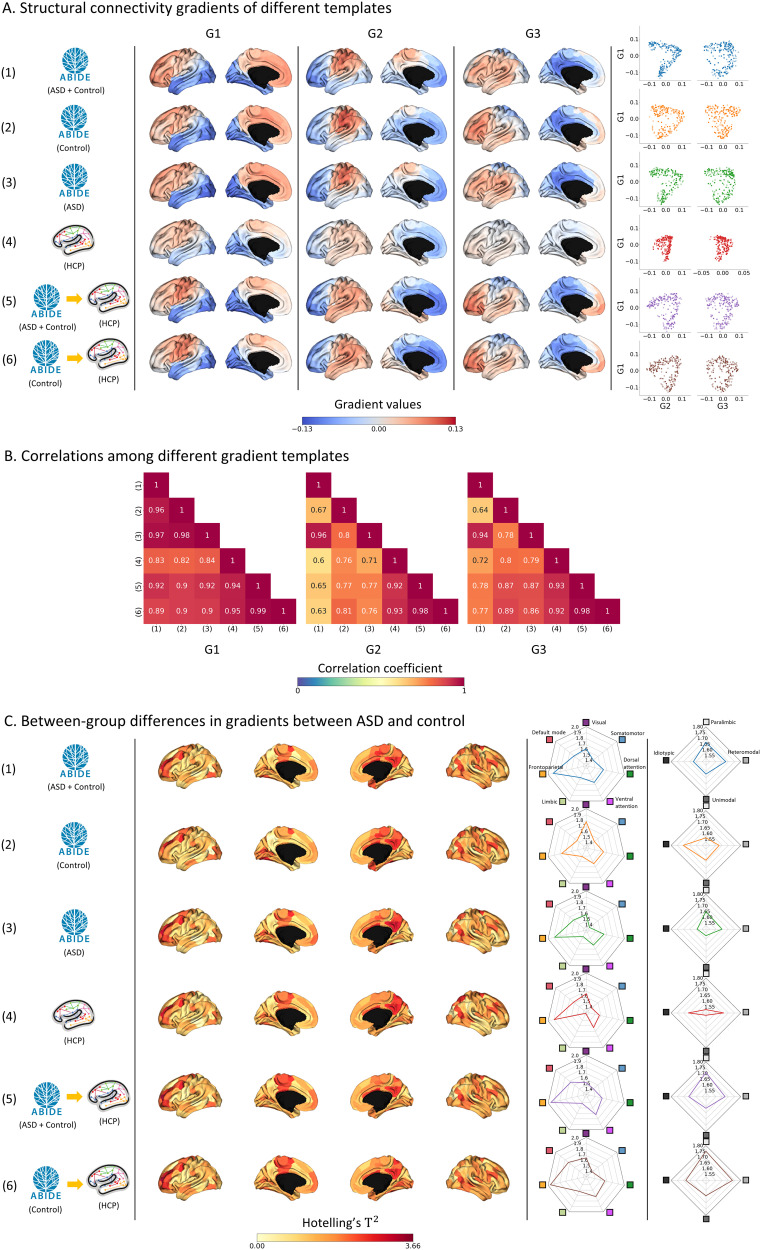
Structural connectivity gradients of different templates and between-group differences. (A) We generated three structural connectivity gradients (G1, G2, and G3) of six different template strategies and plotted them on brain surfaces. The scatterplots display the distribution of gradient pairs. (B) We reported heatmaps of correlation coefficients between different template pairs. The spin permutation test showed that all pairs were significant at the significance level of 0.05. (C) Hotelling’s *T*^2^ statistics of the whole brain are plotted on brain surfaces. We stratified the effects according to seven functional communities and four cortical hierarchical levels using radar plots. Abbreviations: ABIDE, Autism Brain Imaging Data Exchange Initiative; ASD, autism spectrum disorder; HCP, Human Connectome Project.

Similar to the functional gradients, we generated three structural gradients (G1, G2, and G3), which accounted for approximately 47.8% of the information (Supplementary Table 3). G1 showed an anterior–posterior axis, G2 differentiated the sensorimotor and frontal regions, and G3 depicted a lateral-medial axis (Figure 3A). Spatial correlations of the gradient templates showed the highest similarity between Template 5 ASD + control (ABIDE) → HCP and Template 6 control (ABIDE) → HCP (mean ± *SD r* across gradients = 0.98 ± 0.00), and the lowest similarity between Template 1 ASD + control (ABIDE) and Template 4 HCP (*r* = 0.72 ± 0.12; Figure 3B). Even though there were no significant between-group differences in the gradient values (*p*_FDR_ < 0.05), the dorsolateral prefrontal cortex, precuneus, and temporal regions showed strong effects (Figure 3C). The frontoparietal network showed moderate between-group differences when the significance level was relaxed (*p*_FDR_ < 0.1; Supplementary Figure 2). When we stratified the effects according to functional networks (Yeo et al., 2011) and cortical hierarchy (Mesulam, 1998), heteromodal association and paralimbic regions, including the frontoparietal network, showed strong between-group differences. Details on specific between-group differences within functional communities can be found in Supplementary Figure 1B. Overall, in structural connectivity gradients, the effect sizes obtained using the HCP template and its variants (mean ± *SD* statistics = 1.62 ± 0.67 [Template 4]; 1.66 ± 0.64 [Template 5]; 1.69 ± 0.63 [Template 6]; across parcels) were similar to those observed using templates derived from the ABIDE dataset (1.65 ± 0.64 [Template 1]; 1.63 ± 0.64 [Template 2]; 1.62 ± 0.66 [Template 3]). On the other hand, when comparing Templates 4 and 5, more pronounced effects were observed in the paralimbic region in Template 5 (mean ± *SD* statistics = 1.55 ± 0.55 [Template 4]; 1.71 ± 0.55 [Template 5]).

### Replication in a Different Dataset

We conducted a replication study based on a heterogeneous cohort of individuals with focal epilepsy and site-matched controls (for the demographic information, see Supplementary Table 4). Here, the first three functional gradients (G1, G2, and G3) accounted for 48.5% of the information in the input connectivity matrix, and the three structural gradients accounted for 76.7% (Supplementary Figures 3A and 4A). These gradients depicted the same gradual axes as those in the main findings. The spatial patterns of the gradients were consistent across different templates, as determined by 1,000 spin permutation tests with *p* < 0.05 (Supplementary Figures 3B and 4B). Comparing the gradient values between the groups, strong effects were found primarily in the temporal and frontal regions (Supplementary Figures 3C and 4C). Overall, the effects of the HCP template and its variants (i.e., Templates 4, 5, and 6) were consistent with the ASD results (mean ± *SD* statistics = 2.56 ± 1.10 [Template 1]; 2.64 ± 1.07 [Template 2]; 2.69 ± 1.04 [Template 3]; 2.66 ± 1.07 [Template 4]; 2.70 ± 1.08 [Template 5]; 2.72 ± 1.09 [Template 6]; across parcels [functional], 3.86 ± 1.56 [Template 1]; 3.86 ± 1.55 [Template 2]; 3.85 ± 1.56 [Template 3]; 3.93 ± 1.56 [Template 4]; 3.89 ± 1.55 [Template 5]; 3.89 ± 1.55 [Template 6]; across parcels [structural]).

### Sensitivity Analyses

Multiple sensitivity analyses demonstrated the generalizability and robustness of our findings concerning various hyperparameter settings. First, we adopted a cosine similarity kernel instead of a normalized angle when constructing the affinity matrix. Although functional gradients showed similar patterns across the six templates, the structural gradients of the ABIDE-driven and HCP were different in their scales (Supplementary Figures 5 and 6). The results indicate that the normalized angle kernel, which provides an affinity matrix of the normalized scale, may apply to a wide variety of templates compared with the cosine similarity kernel. Second, we evaluated gradient patterns at different spatial scales using Schaefer atlases of 100, 300, and 400 parcels while maintaining other settings constant (Supplementary Figures 7–12). We consistently observed significant between-group differences between the ASD and control groups in the frontoparietal, somatomotor, and attention networks. When we quantitatively compared the between-group effects across spatial granularities by calculating the *T*^2^-statistic maps mapped onto 32k vertices, we observed relatively similar patterns for parcels larger than 200 (Supplementary Figures 13 and 14). Third, when we used the multimodal parcellation scheme (Glasser et al., 2016), we found consistent results with our main findings based on the Schaefer atlas (Schaefer et al., 2018) (Supplementary Figures 15 and 16). Fourth, we also performed functional gradient analysis at the vertex level and found largely consistent results (Supplementary Figure 17). Fifth, we additionally controlled for head motion based on framewise displacement when assessing between-group differences in the gradients. Functional gradients did not show a noticeable difference, but structural gradients showed a decrease in effect size in the visual area (Supplementary Figure 18). Sixth, we compared the between-group difference effects using different group-level templates constructed using subsets of HCP subjects. We found consistent patterns of the effect sizes across different templates with various sample sizes, indicating that the sample size constructing the template gradients did not influence the statistics of the between-group differences (Supplementary Figure 19). Seventh, we investigated the between-group difference effects between the individuals with ASD and neurotypical controls for each site. We found that the between-group difference patterns varied across sites (Supplementary Figures 20–25), highlighting that site-specific factors, such as scanner hardware, participant demographics, and data acquisition protocols, may influence the outcomes of neuroimaging studies. The site-specific findings could be unstable because of the reduced sample size. Indeed, site-specific variations were also reported in previous works using the ABIDE-II dataset (Bayer et al., 2022; He et al., 2020). The observed discrepancies across sites necessitate a cautious interpretation of between-group differences and suggest that future research should aim to understand and, where possible, mitigate the impact of site-specific variables on neuroimaging outcomes. Eighth, when we assessed between-group differences in individuals with ASD and neurotypical controls using adult or children samples, the frontoparietal and attention networks were consistently highlighted, while distinctions were observed in the default mode and visual networks for the functional gradients (Supplementary Figures 26–27). On the other hand, in structural analysis, children showed smaller effects in the limbic/paralimbic areas than adults (Supplementary Figures 28–29). Given the small sample size in adult samples, careful interpretations are required. Lastly, we generated sex-specific gradient templates. Quantitatively comparing the sex-specific HCP templates (i.e., Templates 7 and 8), as well as the aligned templates (i.e., Templates 9 and 10), spatial patterns were largely consistent (Supplementary Figures 30A, 30B and 31A, 31B). The between-group differences in gradient values between the ASD and control groups also showed similar patterns across the brain networks when we compared the results based on the male or female templates (Supplementary Figures 30C and 31C). In sum, the findings suggest that sex does not affect the gradient alignment processes.

### Additional Characterization of the Templates

To assess which template might be a better choice for the gradient studies, we performed classification and prediction analyses. Using the gradients as features, we classified the ASD and control groups using the support vector machine and predicted the Autism Diagnostic Observation Schedule (ADOS) scores using ridge regression. We found that the ABIDE templates aligned to the HCP template showed overall higher performance in both classification and prediction tasks (Supplementary Table 5), suggesting that Template 5 might be useful for the gradient analysis.

## DISCUSSION

The recent adoption of connectome gradient analyses, commonly performed using connectome manifold learning techniques, provides rich information on brain organization and its alterations in clinical conditions. This approach has gained considerable traction in studying the functional (Bethlehem et al., 2020; Cross et al., 2021; Margulies et al., 2016; Mckeown et al., 2020; Murphy et al., 2019; Park, Park, et al., 2021; Yousefi & Keilholz, 2021) and structural (Burt et al., 2018; Masouleh et al., 2020; Paquola, Vos de Wael, et al., 2019; Park, Hong, et al., 2021) organization of the whole brain, as well as specific brain regions (Friedrich et al., 2020; Guell et al., 2018; Lefco et al., 2020; Müller et al., 2020; Royer et al., 2020; Vos de Wael et al., 2018; Wang et al., 2023; Yang et al., 2020) of healthy adults. It has also been used to investigate structure–function coupling (Larivière, Vos de Wael, et al., 2020; Park, Vos de Wael, et al., 2021; Vázquez-Rodríguez et al., 2019) and to explore developmental connectivity changes during typical (Dong et al., 2021; Lau et al., 2021; Paquola, Bethlehem, et al., 2019; Park, Bethlehem, et al., 2021; Xia et al., 2022) and atypical developments (Hong et al., 2019; Park, Hong, et al., 2021). Most of these studies aligned individual gradients using a group-averaged template. Issues with choosing an appropriate template have been raised in previous studies while conducting image registration. Varying brain morphology across different disease states, such as between healthy control subjects and individuals with neurodegenerative disorders (Dadar et al., 2022; Guo et al., 2021), age groups from childhood to the elderly (Fillmore et al., 2015; Sanchez et al., 2012a, 2012b), and ethnicity (Rao et al., 2017; Xie et al., 2015), suggest the necessity for generating population-specific brain templates. Similarly, a technical issue remains in selecting a template for aligning individual gradients. When studying different conditions, for example, neurotypical controls and individuals with ASD, individual gradients were often aligned to the same template gradient constructed using both individuals with ASD and control subjects, even though the gradient patterns were different between the groups. This may lead to misleading findings and further hinder the identification of reliable and robust biomarkers for diseases.

In this study, we systematically compared six template construction strategies based on the ASD control design. Among the six gradient templates, Templates 1, 2, and 3 were defined from the database containing the subjects to be analyzed. Among them, Template 3 included only individuals with ASD, leading to a bias toward atypical over typical development. When comparing functional gradients between individuals with ASD and control subjects, we did not observe the expected differences in somatomotor and default mode areas known to be affected by ASD (Hong et al., 2019). Additionally, idiotypic areas, known to be relevant to ASD (Park, Hong, et al., 2021), were not found in the comparison of the structural gradients. Contrastingly, Template 2 only contained control subjects, while Template 1 included both individuals with ASD and control subjects. Thus, they were relatively free of disease-related biases. Conceptually, considering both groups may reduce bias toward a specific group, but the between-group difference effects were comparable between these two strategies. Based on these findings, we considered an independent HCP dataset. Overall, the effect sizes observed using the HCP dataset template were greater than those observed using the templates derived from the ABIDE dataset. Aligning the gradients of the ABIDE dataset onto this normative HCP data may spread the individual gradients more evenly and increase the effect sizes for assessing between-group differences. We speculate that enhanced effect of HCP may be due to the data quality. Potential factors include the resolution of the imaging data, repetition time (TR), and issues related to gradient nonuniformity. These elements can significantly affect the signal-to-noise ratio and, by extension, the reliability of the detected signals in conventional fMRI studies. Functional connectivity benefits from longer scan times and a higher signal-to-noise ratio of good-quality data, which enhances the stability of correlation measures. Similarly, structural connectivity data are of notably higher quality characterized by acquisitions from a large number of directions. This enhanced data quality inherently stabilizes the axes on the manifold of connectivity patterns. In such a case, a group-level template, possibly derived outside of HCP, is likely to be stable. However, even with high-quality data, it does not mean that individual gradients need not be aligned to the group-level template. When a connectivity matrix of an individual is projected onto a low-dimensional manifold, the axes of individual data might be misaligned from the group-level axes. For example, the first axis of the individual gradient may correspond to the second axis of the group-level gradient, and the direction of the axis may be flipped. Hence, alignment to the group-level template is necessary to evaluate gradient patterns across different subjects. This is necessary regardless of the quality of the target group-level atlas.

Template 4 was derived from the HCP dataset. Using this template revealed additional expected ASD-related alterations in the frontoparietal and attention networks compared with using the ABIDE-driven templates. As discussed above, Template 4, generated using only HCP subjects, does not reflect the age and sex characteristics of the ASD group, which is younger and more male-dominated than the HCP samples. To address this issue, we created Templates 5 and 6, which simultaneously reflect normative and ASD-specific demographic characteristics. We created Template 5 using all ABIDE subjects (individuals with ASD and control subjects), and the group template was aligned to the HCP template. Therefore, this template has the properties of both Templates 1 and 4. It retained ASD-specific demographics while benefiting from the advantages of the HCP template. When comparing Templates 4 (HCP) and 5 (ASD + control [ABIDE] → HCP), we observed diminished effects in the default mode regions for functional gradients and paralimbic areas for structural gradients in Template 5. Using functional gradients, a previous study found that default mode regions had stronger effects in adults with ASD than in children with ASD (Hong et al., 2019). Another study using structural gradients found that adults with ASD had stronger effects in the paralimbic areas than children with ASD (Park, Hong, et al., 2021). These findings suggest age-related differences in ASD connectopathy. Our study subjects were primarily children; thus, a tendency toward reduced effects in these transmodal regions when using the Template 5 may be more reliable than when using Template 4. On the other hand, Template 6 was created by starting with only control subjects in ABIDE and aligning the group template to the HCP template, giving it the properties of both Templates 2 and 4. This template fared well in detecting regions for functional connectomes but resulted in more control-like effects for structural connectomes.

Our replication analysis of individuals with focal epilepsy demonstrated the robustness of our findings across different disease states. Epilepsy is characterized by recurrent seizures occurring in specific brain regions, such as the temporal, frontal, or occipital lobes, and has been shown to affect not only seizure focus but also large-scale brain networks (Gleichgerrcht et al., 2015; Hatton et al., 2020; Larivière, Bernasconi, et al., 2021; Larivière, Rodríguez-Cruces, et al., 2020; Park et al., 2022; Royer, Bernhardt, et al., 2022; Sisodiya et al., 2022; Stasenko et al., 2022; Tavakol et al., 2019). Here, we found that the HCP-aligned Templates 4, 5, and 6 had increased effects compared with the study subject–based Templates 1, 2, and 3. Similar to the individuals with ASD studied in our main analyses, the study subjects of individuals with epilepsy were heterogeneous and contained multiple seizure foci. As such, the gradient alignment strategy proposed in our study may be suitable for retrospective studies investigating global shifts in brain network organization in multiple disease conditions.

We validated the consistency of our findings using vertex-level data, multiple spatial scales of the Schaefer atlas (Schaefer et al., 2018), and an independent multimodal parcellation-based atlas (Glasser et al., 2016). As noted in the previous work, selecting atlases with varying spatial scale, coverage, and shape may influence the outcome of a given study (Revell et al., 2022). They suggested a framework for determining which atlas to use in the study with three aspects: (a) descriptive, (b) explanatory, and (c) predictive validity. Descriptive validity indicates that the atlas should include features relevant to the study, and explanatory and predictive validities represent whether the study hypothesis is about explaining causality or making predictions. If the study outcomes are robust across different atlases, it becomes strong evidence to support the claims of the study. In general, researchers should carefully consider the selection of brain atlases and validate their findings using various types of atlases.

Together, considering the various strategies for generating gradient templates, the study dataset-derived and HCP-aligned Template 5 may be applicable for general use, because it retains the advantages of comprehensive modeling for the demographics of study subjects (hence avoiding bias toward diseased cases) as well as the normative population, and furthermore showed good performance in disease classification and symptom prediction. Template 5, which features enhanced normal population modeling and correction for a specific dataset, may potentially provide more reliable and robust results for both functional and structural gradients across different study settings. However, if the subject demographics are well-matched to those of the HCP, using Template 4 could be a good option. Also, aligning the individual gradients to a different template may yield varying results. Because of this, the target template should be carefully chosen to accommodate the design of the study.

In this study, by comparing six group-level templates, we found that using a template combining enhanced normal population modeling from HCP and group-specific characteristics from study subjects might be a good option for studying connectome gradients. Our study contributes to better gradient analysis by emphasizing the importance of selecting an appropriate template for aligning individual gradients. We highlight the critical role of selecting an appropriate group-level template for Procrustes rotation in identifying group differences in gradient patterns. Template selection and subsequent alignment ensure that individual data points correctly align with group-level axes, thereby maximizing group differences and allowing for accurate, meaningful interpretations. Additionally, we underscore the vulnerability of existing gradient approaches and recommend a reasonable group-level template to use. It is very important to ensure the reliability and robustness of the findings because using an inappropriate template can lead to inaccurate results and hinder the identification of reliable biomarkers for atypical brain conditions.

Several issues need to be investigated in future studies. Our experiments were confined to comparing ASD or focal epilepsy with neurotypical controls, and the findings should be replicated under other imaging parameter choices. Additionally, unlike functional gradients, methods for generating structural connectomes have not yet been standardized. In this study, we adopted an existing approach to construct the gradients of the structural connectome (Park, Hong, et al., 2021; Park, Vos de Wael, et al., 2021). Further research is required to unveil the topological and biological underpinnings of the structural gradients. Our findings were replicated in an independent heterogeneous cohort of individuals with focal epilepsy and site-matched controls. Still, further validation is necessary to determine whether our findings will hold for different diseases. Additionally, we found that the between-group difference effects between individuals with ASD and neurotypical controls vary across sites. Thus, future studies should consider strict site correction methods, such as site regression, Combat (Fortin et al., 2018), or other approaches. Lastly, we aligned individual gradients to the HCP template despite differences in the demographic information across datasets. Further studies need to consider different age distributions when constructing gradient templates for better alignment. Taken together, our findings provide a basis for future connectome gradient studies.

## METHODS

### Participants

#### ABIDE dataset.

The ABIDE-II database provides a large number of samples collected from multiple centers. Among them, 115 subjects were chosen based on the following criteria: (a) complete multimodal imaging data, that is, T1-weighted MRI, resting-state functional MRI (rs-fMRI), and diffusion MRI with sufficient MRI data quality (i.e., scanned with 3 T scanner); (b) acceptable cortical surface extraction; (c) low head motion in the rs-fMRI time series (<0.3 mm framewise displacement); (d) sites with at least 10 individuals with ASD and 10 neurotypical controls; and (e) sites provided demographic (age and sex) and clinical scores (ADOS). Finally, subjects were obtained from three different sites: New York University Langone Medical Center, Trinity College Dublin, and Institut Pasteur and Robert Debré Hospital. Details are described in Supplementary Table 1. ABIDE data collection was performed under the local Institutional Review Board guidelines. All ABIDE datasets were anonymized, and no protected health information was included, following the Health Insurance Portability and Accountability Act guidelines and the 1000 Functional Connectomes Project or Instrument Neutral Distributed Interface protocols.

#### HCP dataset.

We also considered an additional control dataset of multimodal MRI data from the HCP database (Van Essen, Ugurbil, et al., 2012). From the S1200 release of the HCP Young Adult cohort, we excluded subjects who did not complete full multimodal imaging (i.e., T1- and T2-weighted MRI, rs-fMRI, and diffusion MRI) and had family relationships (i.e., twins), resulting in 479 subjects (mean ± *SD* age = 28.18 ± 3.93 years; 52.19% male; Supplementary Table 1). All MRI data used in this study were publicly available and anonymized. Subject recruitment procedures and informed consent forms, including consent to share de-identified data, were approved by the Washington University Institutional Review Board as part of the HCP.

#### Epilepsy dataset.

For replication of our findings, an independent dataset was obtained from the Montreal Neurological Institute and Hospital. The dataset contained 47 healthy controls (age = 33 ± 4.70 years; 46.8% male), many of whom were previously included in an open-access data release (Royer, Rodríguez-Cruces, et al., 2022), as well as 48 patients with drug-resistant focal epilepsy (age = 34 ± 10.62 years; 45.8% male; Supplementary Table 2). Individuals with epilepsy were diagnosed with temporal lobe epilepsy (*n* = 34), frontal lobe epilepsy (*n* = 5), or other complex types of epilepsy such as temporo-occipital and frontal-opercular epilepsy (*n* = 9). This heterogeneous characteristic of the disease matches well with the heterogeneity of the ASD dataset. There were no significant differences in age and sex between individuals with focal epilepsy and healthy controls (age: *t* = −0.60, *p* = 0.55; sex: *χ*^2^ = 0.01, *p* = 0.92). After a thorough examination that included a detailed clinical history, a neurological examination, a review of medical records, video-electroencephalography recordings of ictal and interictal events, and a clinical MRI evaluation, the International League Against Epilepsy criteria were used to diagnose epilepsy subtypes and lateralize seizures.

### MRI Acquisition

#### ABIDE dataset.

ABIDE data were acquired as follows: At the New York University Langone Medical Center site, multimodal imaging data were acquired using a 3 T Siemens Allegra scanner. T1-weighted data were obtained using a 3D magnetization-prepared rapid acquisition gradient echo (MPRAGE) sequence (repetition time [TR] = 2,530 ms; echo time [TE] = 3.25 ms; inversion time [TI] = 1,100 ms; flip angle = 7°; matrix = 256 × 192; and voxel size = 1.3 × 1.0 × 1.3 mm^3^). The rs-fMRI data were acquired using a 2D echo planar imaging (EPI) sequence (TR = 2,000 ms; TE = 15 ms; flip angle = 90°; matrix = 80 × 80; number of volumes = 180; and voxel size = 3.0 × 3.0 × 4.0 mm^3^). The diffusion MRI was scanned using a 2D spin-echo EPI (SE-EPI) sequence (TR = 5,200 ms; TE = 78 ms; matrix = 64 × 64; voxel size = 3 mm, isotropic; 64 directions; b-value = 1,000 s/mm^2^; 1 b0 image). A 3 T Philips Achieva scanner was used to collect imaging data at the Trinity College Dublin site. T1-weighted MRI scans were acquired using a 3D MPRAGE sequence (TR = 8,400 ms; TE = 3.90 ms; TI = 1,150 ms; flip angle = 8°; matrix = 256 × 256; voxel size = 0.9 mm isotropic). The rs-fMRI data were acquired using a 2D EPI (TR = 2,000 ms; TE = 27 ms; flip angle = 90°; matrix = 80 × 80; number of volumes = 210; and voxel size = 3.0 × 3.0 × 3.2 mm), and diffusion MRI data were acquired using a 2D SE-EPI (TR = 20,244 ms; TE = 79 ms; matrix = 124 × 124; voxel size = 1.94 × 1.94 × 2 mm^3^; 61 directions; b-value = 1,500 s/mm^2^; and 1 b0 image). For the Institut Pasteur and Robert Debré Hospital site, MRI data were scanned using a 1.5 T Philips Achieva scanner. T1-weighted data were acquired using a 3D MPRAGE sequence (TR = 25 ms; TE = 5.6 ms; flip angle = 30°; matrix = 240 × 240; voxel size = 1 mm isotropic). The rs-fMRI data were acquired using a 2D EPI (TR = 2,700 ms; TE = 45 ms; flip angle = 90°; matrix = 64 × 63; number of volumes = 85; and voxel size = 3.59 × 3.65 × 4 mm), and diffusion MRI data were acquired using a 2D EPI sequence (TR = 5,407 ms; TE = 86 ms; matrix = 240 × 190; voxel size = 2.5 mm isotropic; 32 directions; b-value = 1,000 s/mm^2^; and 1 b0 image).

#### HCP dataset.

HCP data were collected using a 3 T Siemens Skyra scanner. T1-weighted data were acquired using a 3D MPRAGE sequence (TR = 2,400 ms; TE = 2.14 ms; matrix = 224 × 224; voxel size = 0.7 mm isotropic, 256 slices). The rs-fMRI data were acquired using gradient-echo EPI (TR = 720 ms; TE = 33.1 ms; matrix = 208 × 180, voxel size = 2 × 2 × 2 mm^3^; 72 slices). Finally, diffusion MRI data were acquired using SE-EPI (TR = 5,520 ms; TE = 89.5 ms; matrix = 210 × 180; voxel size = 1.25 mm isotropic; 270 directions; b-value = 1,000/2,000/3,000 s/mm^2^; and 18 b0 images).

#### Epilepsy dataset.

Epilepsy data were collected using a 3 T Siemens Magnetom Prisma-Fit scanner equipped with a 64-channel head coil. Two T1-weighted scans were obtained using a 3D MPRAGE sequence (TR = 2,300 ms; TE = 3.14 ms; TI = 900 ms; flip angle = 9°; matrix = 320 × 320; iPAT = 224 slices; voxel size = 0.8 mm^3^). The rs-fMRI data were collected with a 2D-BOLD EPI (TR = 600 ms; TE = 30 ms; flip angle = 52°; matrix = 240 × 240; number of volumes = 700; multi-band factor = 6; echo spacing = 0.54 ms; 48 slices; and voxel size = 3 mm^3^), and the diffusion MRI data were obtained using a 2D SE-EPI sequence (TR = 3,500 ms; TE = 64.4 ms; matrix = 224 × 224; voxel size = 1.6 × 1.6 × 1.6 mm^3^; flip angle = 90°; matrix = 224 × 224; echo spacing = 0.54 ms; three shells with b-values 300/700/2,000 s/mm^2^, and 10/40/90 directions; and three b0 images).

### Data Preprocessing

#### ABIDE dataset.

The T1-weighted data of the ABIDE dataset were processed using FreeSurfer (Dale et al., 1999; Fischl, 2012; Fischl et al., 2001; Fischl, Sereno, & Dale, 1999; Fischl, Sereno, et al., 1999; Segonne et al., 2007). The preprocessing steps consisted of gradient nonuniformity correction, non-brain tissue removal, intensity normalization, and tissue segmentation. White and pial surfaces were generated through triangular surface tessellation, topology correction, inflation, and spherical registration to the fsaverage template. The T1-weighted data quality was visually assessed, and data with incorrect cortical segmentation were removed. The ABIDE database provided preprocessed rs-fMRI data (https://preprocessed-connectomes-project.org/abide/), which were processed using a Configurable Pipeline for the Analysis of Connectomes (C-PAC; https://fcp-indi.github.io; Craddock et al., 2013). The pipeline included slice timing and head motion correction, skull stripping, and intensity normalization. CompCor was used to remove nuisance variables such as head motion, average white matter, cerebrospinal fluid signals, and linear and quadratic trends (Behzadi et al., 2007). The noise-removed rs-fMRI data were coregistered to the T1-weighted data in MNI152 space using boundary-based rigid-body and nonlinear transformations. Volumetric rs-fMRI data were mapped to subject-specific mid-thickness surfaces and resampled to the Conte69 surface. Finally, surface-based spatial smoothing was applied with a full width at a half maximum (FWHM) of 5 mm. Data with framewise displacements greater than 0.3 mm were excluded. Diffusion MRI data were preprocessed using MRtrix3 (Tournier et al., 2019), which includes corrections for susceptibility distortions, head movement, and eddy currents. Anatomically constrained tractography was performed using different tissue types derived from T1-weighted MRI, including cortical and subcortical gray matter, white matter, and cerebrospinal fluid (Smith et al., 2012). T1-weighted data were registered to the diffusion MRI data based on boundary-based registration, and transformation was applied to different tissue types to register them onto the native diffusion MRI space. Multishell and multi-tissue response functions were estimated (Christiaens et al., 2015), and constrained spherical deconvolution and intensity normalization were conducted (Jeurissen et al., 2014). The tractogram was built using a probabilistic method (Tournier et al., 2010, 2012, 2019) with 40 million streamlines, with a maximum tract length of 250 and a fractional anisotropy cutoff of 0.06. Spherical deconvolution-informed filtering of tractograms (SIFT2) was employed to determine the optimum cross-section multiplier for each streamline (Smith et al., 2015), and the whole-brain streamlines weighted by the cross-section multipliers were reconstructed.

#### HCP dataset.

The HCP database provides minimally preprocessed multimodal MRI data (Glasser et al., 2013). The structural MRI data were corrected for gradient nonlinearity and b0 distortions. T1- and T2-weighted data were coregistered with rigid-body transformation, and the bias field was adjusted with the inverse intensities from the T1- and T2-weighting. After registration of the processed data onto the MNI152 space, white and pial surfaces were generated following the boundaries between different tissues (Dale et al., 1999; Fischl, Sereno, & Dale, 1999; Fischl, Sereno, et al., 1999). A mid-thickness surface was generated by averaging the white and pial surfaces, and an inflated surface was generated. Using MSMAll, the spherical surface with 164k vertices was aligned to the Conte69 template (Robinson et al., 2014), and then the mesh was scaled down to 32k vertices. The rs-fMRI data underwent distortion and head motion and magnetic field bias corrections, skull removal, intensity normalization, and registration to the MNI152 space (Glasser et al., 2016; Van Essen, Glasser, et al., 2012). Noise components attributed to head movement, white matter, cardiac pulsation, and arterial and large vein-related contributions were removed using FMRIB’s independent component analysis–based X-noiseifier (FIX; Salimi-Khorshidi et al., 2014). The preprocessed fMRI data were mapped to the standard grayordinate space using a cortical ribbon-constrained volume-to-surface mapping algorithm. The total mean of the time series of each left-to-right or right-to-left phase-encoded dataset was subtracted to adjust the discontinuity between the two datasets, and they were concatenated to form single time series data. Diffusion MRI data underwent b0 intensity normalization and correction for susceptibility distortion, eddy currents, and head movement. Diffusion tractography was performed using the same procedure as that used for the ABIDE dataset.

#### Epilepsy dataset.

Micapipe (https://github.com/MICA-MNI/micapipe; Rodríguez-Cruces et al., 2022), a comprehensive open-source multimodal MRI processing pipeline integrating multiple preprocessing software of FreeSurfer, FSL, AFNI, MRtrix3, ANTs, and workbench (Avants et al., 2011; Cox, 1996; Fischl, 2012; Jenkinson et al., 2012; Marcus et al., 2011; Tournier et al., 2019), was used to preprocess the data. T1-weighted data were de-obliqued and reoriented to the left-posterior-inferior orientation before being linearly coregistered and averaged. The resulting images were intensity nonuniformity-corrected (Tustison et al., 2010), intensity-normalized, and skull-stripped. Subcortical structures were segmented using FSL FIRST (Patenaude et al., 2011), and cortical surfaces were generated using FreeSurfer (Dale et al., 1999; Fischl, 2012; Fischl et al., 2001; Fischl, Sereno, & Dale, 1999; Fischl, Sereno, et al., 1999; Segonne et al., 2007). The rs-fMRI data were preprocessed as follows: The first five volumes were discarded to ensure magnetic field saturation, and the images were reoriented. Head motion correction was performed by registering all volumes to the mean volume across time. Distortion correction was performed using main- and reverse-phase field maps. The nuisance variables were removed using FMRIB’s FIX (Salimi-Khorshidi et al., 2014) and by regressing the time points identified as motion outliers (i.e., motion spike regression). Processed volumetric data were registered to the native FreeSurfer space using boundary-based registration (Greve & Fischl, 2009) and mapped to individual surface models using trilinear interpolation. The rs-fMRI data on the native surface were spatially smoothed with a full width at a half maximum of 10 mm, registered to the Conte69 template with 164k vertices using MSMAll (Robinson et al., 2014), and downsampled to a 10k vertex mesh. The diffusion MRI data were denoised (Veraart et al., 2016), b0 intensity normalized (Tustison et al., 2010), and corrected for susceptibility distortion, head motion, and eddy currents using reverse-phase encoding data. Diffusion tractography was performed using the same procedure described above.

### Group-Level Template Gradient Construction

We constructed cortex-wide functional and structural connectomes using the Schaefer atlas with 200 parcels (Schaefer et al., 2018). A functional connectivity matrix was constructed based on Pearson’s correlations of the functional time series between two different regions for each individual. The correlation coefficients were Fisher’s r-to-z-transformed at the subject-specific individual level, and the group-level connectivity matrix was defined by averaging individual z-transformed connectivity matrices. The structural connectome was defined based on diffusion tractography, and a structural connectivity matrix was constructed using the number of weighted streamlines between two different regions for each individual. The individual subject-level structural connectivity matrix was log-transformed to minimize the connectivity strength variance. The group-level connectivity matrix was defined using a distance-dependent thresholding method that retains the edge-length distance connections of individuals (Betzel et al., 2019) and was log-transformed.

We estimated the functional and structural gradients using the BrainSpace toolbox (https://github.com/MICA-MNI/BrainSpace; Vos de Wael et al., 2020). For functional connectivity, the group-level connectivity matrix was thresholded to retain the top 10% of elements per row. We used a normalized angle kernel to compute an affinity matrix that captured the interregional similarity in connection patterns between cortical regions. Instead of cosine similarities ranging from −1 to 1, we employed a normalized angle kernel to circumvent negative similarities. A normalized angle converts the cosine similarity from 0 to 1, where 1 indicates equal angles and 0 represents opposite angles. Next, we employed diffusion map embedding (Coifman & Lafon, 2006), a nonlinear manifold learning technique, to estimate the low-dimensional eigenvectors (i.e., gradients). This approach places interconnected brain regions close together in the newly defined manifold space and weakly connected regions farther away. For structural connectivity, a normalized angle kernel with no thresholding was employed, as the structural connectome was already sparse, and the gradients were estimated via diffusion map embedding. In the structural connectome, gradients are estimated by treating the left and right hemispheres separately to address the lateralization problem (Park, Bethlehem, et al., 2021). At the level of the individual, connectome gradients were calculated in the same way as group-level gradients, except for generating the group representative matrix.

We generated three cortex-wide template gradients (G1, G2, and G3) for both functional and structural connectivity data to maintain the same cortical axes as those found in previous studies (Margulies et al., 2016; Park, Hong, et al., 2021). We compared different group-level template construction strategies as follows: (1) mixing ASD and neurotypical controls from ABIDE, noted as ASD + control (ABIDE); (2) using only controls from ABIDE, noted as control (ABIDE); (3) using only ASD from ABIDE, noted as ASD (ABIDE); (4) using only controls from HCP, noted as HCP; (5) aligning group templates of 1 to 4, noted as ASD + control (ABIDE) → HCP; and (6) aligning group templates of 2 to 4, noted as control (ABIDE) → HCP. The gradients were aligned via Procrustes rotation (Langs et al., 2015; Vos de Wael et al., 2020). The HCP template is an independent dataset, and aligning the ABIDE templates onto the HCP is an attempt to mitigate the bias and demographic characteristics of the ABIDE dataset.

### Statistical Analyses and Assessing the Reliability of Different Templates

To examine the similarities among the different templates, we calculated the spatial correlations of each template gradient (G1, G2, and G3) for both functional and structural connectomes. For each possible pair, we compared two different templates. The statistical significance of the correlations was evaluated using 1,000 nonparametric spin permutation tests to preserve spatial autocorrelations (Alexander-Bloch et al., 2018; Larivière, Paquola, et al., 2021). This test considers the inherent spatial smoothness of the brain maps and compares the actual correlation coefficient and *p* value with a null distribution generated by an ensemble of correlation coefficients of spatially permuted surface maps.

To assess the reliability of the gradient templates, we evaluated the effect sizes of between-group differences in gradient values between individuals with ASD and neurotypical controls. To this end, we aligned the gradients of individual subjects to six different templates using Procrustes rotation (Langs et al., 2015). We then fitted cortex-wide multivariate linear models to compare aligned gradients between individuals with ASD and controls, as implemented in the BrainStat toolbox (Larivière et al., 2023), controlling for age, sex, and site. The linear model was *Y* = *intercept* + *group* + *age* + *sex* + *site*, where age, sex, and site were the fixed effects. The model was fitted using three gradients. We estimated the magnitude of the between-group difference effects using Hotelling’s *T*^2^ statistic to compare the effect sizes of the different templates (Larivière et al., 2023; Worsley et al., 2009). Multiple comparisons across brain regions were corrected using the false discovery rate (FDR) procedure (Benjamini & Hochberg, 1995).

### Replication Using an Independent Dataset of Focal Epilepsy

We repeated the analyses of generating and aligning gradients with six different strategies to construct group-level templates using an independent dataset of healthy controls and individuals with focal epilepsy obtained from the Montreal Neurological Institute and Hospital (Royer, Rodríguez-Cruces, et al., 2022). Subjects with left temporal lobe epilepsy were hemisphere-flipped to match the seizure foci (Park et al., 2022).

### Sensitivity Analyses

Multiple sensitivity analyses were conducted to examine the robustness of our findings. First, we used a cosine similarity kernel instead of a normalized angle to construct an affinity matrix. Second, we evaluated different spatial scales of 100, 300, and 400 parcels. Third, we performed the same analysis using multimodal parcellation (Glasser et al., 2016) to assess whether the findings can be replicated with different types of parcellation schemes. Fourth, we also evaluated our findings using vertex-level data. Fifth, we compared the gradient values between the ASD and control groups, considering the influence of head motion measured by framewise displacement as an additional covariate in the analysis. The framewise displacement was estimated from rs-fMRI and diffusion MRI to control head motion effects from functional and structural gradients, respectively. Sixth, we randomly selected subsets from the HCP data (100, 200, 300, and 400 subjects) to create the group-level templates. We evaluated the between-group effects between individuals with ASD and neurotypical controls by changing the group-level templates. Seventh, we conducted site-specific analyses to further explore the effects of the site on the between-group differences. In this case, only age and sex were considered in the linear model as covariates. Eighth, to assess the age-related effects of individuals with ASD, we conducted separate analyses for children (age < 18, *n* = 80 [ASD = 48, control = 32]) and adults (age ≥ 18, *n* = 35 [ASD = 24, control = 11]) within the ABIDE cohort. Lastly, we generated sex-specific HCP templates using male and female participants separately to evaluate the effect of potential sex effect. Specifically, we generated gradient Template 7 composed solely of males (HCP Male) and Temmplate 8 composed solely of females (HCP Female). Additionally, we created Template 9, ASD + control (ABIDE) → HCP Male, and Template 10, ASD + control (ABIDE) → HCP Female, by aligning the ABIDE (ASD + control) group template to Templates 7 and 8, respectively.

### Classification and Prediction Analyses

To evaluate the clinical validity of the gradient templates, we performed additional analyses of (a) classifying ASD and controls and (b) predicting the ADOS scores. We used the gradients (i.e., G1 + G2 + G3 = 600 features) as well as age, sex, and site information as covariates. We applied the support vector machine for the classification task and ridge regression for the prediction task. The classification and prediction were performed based on the fivefold cross-validations, where four out of five splits of the data were used for training, and the one partition left was used for validation. We repeated the analysis 100 times with different training and test sets to avoid subject selection bias.

## SUPPORTING INFORMATION

Supporting information for this article is available at https://doi.org/10.1162/netn_a_00382.

## AUTHOR CONTRIBUTIONS

Sunghun Kim: Conceptualization; Data curation; Formal analysis; Methodology; Software; Validation; Visualization; Writing – original draft. Seulki Yoo: Methodology; Resources; Validation; Writing – review & editing. Ke Xie: Data curation; Formal analysis; Resources; Writing – review & editing. Jessica Royer: Data curation; Formal analysis; Resources; Writing – review & editing. Sara Larivière: Data curation; Formal analysis; Resources; Writing – review & editing. Kyoungseob Byeon: Methodology; Resources; Writing – review & editing. Jong Eun Lee: Investigation; Writing – review & editing. Yeongjun Park: Data curation; Resources. Sofie L. Valk: Conceptualization; Writing – review & editing. Boris C. Bernhardt: Conceptualization; Funding acquisition; Methodology; Resources; Software; Writing – review & editing. Seok-Jun Hong: Methodology; Writing – review & editing. Hyunjin Park: Conceptualization; Data curation; Formal analysis; Funding acquisition; Investigation; Methodology; Project administration; Resources; Software; Supervision; Validation; Visualization; Writing – original draft. Bo-yong Park: Conceptualization; Data curation; Formal analysis; Funding acquisition; Investigation; Methodology; Project administration; Resources; Software; Supervision; Validation; Visualization; Writing – original draft.

## FUNDING INFORMATION

Bo-yong Park, National Research Foundation of Korea (https://dx.doi.org/10.13039/501100003725), Award ID: NRF-2022R1A5A7033499. Bo-yong Park, Institute for Information and Communications Technology Promotion (https://dx.doi.org/10.13039/501100010418), Award ID: 2022-0-00448/RS-2022-II220448, Deep Total Recall: Continual Learning for Human-Like Recall of Artificial Neural Networks. Bo-yong Park, Institute for Information and Communications Technology Promotion (https://dx.doi.org/10.13039/501100010418), Award ID: RS-2022-00155915, Artificial Intelligence Convergence Innovation Human Resources Development (Inha University). Hyunjin Park and Bo-yong Park, Institute for Information and Communications Technology Promotion (https://dx.doi.org/10.13039/501100010418), Award ID: RS-2021-II212068, Artificial Intelligence Innovation Hub. Hyunjin Park and Bo-yong Park, Institute for Basic Science, Award ID: IBS-R015-D1. Boris C. Bernhardt, Institut canadien d’information sur la santé (https://dx.doi.org/10.13039/100030898), Award ID: FDN-154298 and PJT-174995. Boris C. Bernhardt, SickKids Foundation, Award ID: NI17-039. Boris C. Bernhardt, Natural Sciences and Engineering Research Council, Award ID: Discovery-1304413. Boris C. Bernhardt, Centre Azrieli de recherche sur l’autisme, Institut et Hôpital Neurologiques de Montréal (https://dx.doi.org/10.13039/100019715), Award ID: ACAR. Boris C. Bernhardt, BrainCanada, Award ID: BrainCanada. Boris C. Bernhardt, Fonds de Recherche du Québec – Santé (https://dx.doi.org/10.13039/501100000156), Award ID: Fonds de la Recherche du Québec – Santé. Boris C. Bernhardt, Helmholtz International BigBrain Analytics and Learning Laboratory, Award ID: Hiball. Boris C. Bernhardt, Canada Research Chairs Program, Award ID: CRC. Hyunjin Park, National Research Foundation of Korea, Award ID: NRF-2020M3E5D2A01084892. Hyunjin Park, AI Graduate School Support Program, Award ID: RS-2019-II190421. Hyunjin Park, ICT Creative Consilience Program, Award ID: RS-2020-II201821.

## DATA AVAILABILITY

The imaging and phenotypic data were provided, in part, by the Human Connectome Project, WU-Minn Consortium (https://www.humanconnectome.org/), and by the Autism Brain Imaging Data Exchange Initiative (ABIDE-II; https://fcon_1000.projects.nitrc.org/indi/abide/). The template gradients used in this study are available at https://github.com/CAMIN-neuro/caminopen/tree/master/gradient_align (Park, 2023).

## CODE AVAILABILITY

The codes for gradient generation are available at https://github.com/MICA-MNI/BrainSpace, and those for multivariate analysis are available at https://github.com/MICA-MNI/BrainStat.

## Supplementary Material



## TECHNICAL TERMS

Functional connectivity: Statistical relationships between brain regions calculated based on the time series correlation of functional activity.
Structural connectivity: Physical connections between different brain regions, typically defined using diffusion MRI-based tractography to map white matter streamlines.
Gradient: A representation that maps a continuous spectrum of connectivity patterns within the brain.
Manifold learning: A machine learning technique to discover lower dimensional structures within high-dimensional data.
Spin permutation: A statistical method used to assess the significance of spatial patterns by rotating brain features across a spherical space.
Multivariate analysis: A statistical technique used to analyze data involving multiple variables at once to identify associations among these variables simultaneously.
Gradient alignment: The process of matching gradients across different datasets to enable meaningful comparisons and analyses of brain organization patterns.
Support vector machine: A supervised machine learning method that finds the optimal hyperplane to separate different classes with maximum margin in a high-dimensional space.
Ridge regression: A type of linear regression that includes a regularization term to prevent overfitting by penalizing large coefficients.

## Supporting Information

https://github.com/CAMIN-neuro/caminopen/tree/master/gradient_align
