# Supplementary material for: Comparison of different group-level templates in gradient-based multimodal connectivity analysis
Source: Netw Neurosci. 2024 Dec 10;8(4):1009–31. doi: 10.1162/netn_a_00382 (PMC11674319; doi:10.1162/netn_a_00382)
Supplement: Supplementary file 1 [file netn-8-4-1009-s001.pdf]

SUPPLEMENTARY INFORMATION

Supplementary Table 1 | Demographic information of the ABIDE and HCP datasets.

**Information**		**ABIDE**						**p-value**	**HCP**
		**NYU**		**TCD**		**IP**			
**Number**	**ASD**	29		18		12		0.138	479
	**Control**	18		19		19			
**Age (years) (mean ± SD)**	**ASD**	9.61 ± 6.16	p=0.807	14.46 ± 3.30	p=0.209	15.80 ± 5.72	p=0.011	0.001	28.18±3.93
	**Control**	10.01 ± 3.95		15.83 ± 3.21		23.84 ± 9.10			
**Sex (male:female)**	**ASD**	24:5	p=0.384	18:0	p=1	9:3	p=0.138	<0.001	250:229
	**Control**	17:1		19:0		8:11			

Cohort-specific demographic information, including the number of subjects, age at scan (in years), and sex, is described.

The p-values for age were derived based on the two-sample t-test or ANOVA between groups or sites. Those for the number of subjects and sex were based on Fisher's exact test.

Abbreviations: ABIDE, Autism Brain Imaging Data Exchange; NYU, New York University Langone Medical Center; TCD, Trinity College Dublin; IP, Institut Pasteur and Robert Debré Hospital; HCP, Human Connectome Project; ASD, autism spectrum disorder; SD, standard deviation.

Supplementary Table 2 | Explained information for each functional gradient across different templates

Functional	G1	G2	G3	Total
Template (1)	17.65%	18.63%	11.02%	47.30%
Template (2)	17.75%	18.48%	11.22%	47.45%
Template (3)	17.62%	18.77%	10.73%	47.12%
Template (4)	20.47%	18.44%	13.62%	52.53%

Templates (5) and (6) have the same explained information as templates (1) and (2) because they are the templates (1) and (2) aligned to the HCP, respectively.

Supplementary Table 3 | Explained information for each structural gradient across different templates

Structural	LH				RH			
	G1	G2	G3	Total	G1	G2	G3	Total
Template (1)	18.49%	15.42%	13.67%	47.58%	18.72%	15.78%	13.50%	48.00%
Template (2)	17.79%	15.91%	14.13%	47.83%	17.67%	15.02%	15.36%	48.05%
Template (3)	18.73%	14.67%	13.86%	47.26%	18.56%	14.86%	13.94%	47.36%
Template (4)	37.99%	27.87%	9.09%	74.95%	40.17%	22.29%	11.69%	74.15%

Templates (5) and (6) have the same explained information as templates (1) and (2) because they are the templates (1) and (2) aligned to the HCP, respectively. Abbreviations: LH, left hemisphere; RH, right hemisphere.

Supplementary Table 4 | Demographic information of the epilepsy dataset.

Information	Focal Epilepsy				Control	p-value
	TLE	FLE	Others	p-value		
**Number**	34 (L = 24, R = 10)	5	9	-	47	-
**Age (years) (mean ± SD)**	35 ± 10.40	31 ± 9.26	32 ± 12.62	0.641	33 ± 4.70	0.549
**Sex (male:female)**	16:18	3:2	3:6	0.656	22:25	0.924

The demographic information of the number of subjects, age at scan, and sex according to the type of epilepsy are described.

The p-values for age were derived based on the Kruskal-Wallis or two-sample t-test between the groups, and those for sex were based on Fisher's exact or chi-squared test.

Abbreviations: TLE, temporal lobe epilepsy; FLE, frontal lobe epilepsy; SD, standard deviation; L, left; R, right

Supplementary Table 5 | Classification and prediction performance using gradients

	**Functional gradients**		**Structural gradients**	
	Classification Accuracy	Regression Mean absolute error	Classification Accuracy	Regression Mean absolute error
**Template (1)**	0.601±0.032	**3.598±0.193**	0.498±0.032	**3.266±0.184**
**Template (2)**	0.615±0.032	3.661±0.196	0.500±0.027	**3.176±0.173**
**Template (3)**	0.605±0.032	3.647±0.191	**0.513±0.030**	3.312±0.184
**Template (4)**	**0.624±0.033**	**3.627±0.179**	0.502±0.032	**3.197±0.176**
**Template (5)**	**0.624±0.034**	**3.643±0.184**	**0.512±0.035**	3.355±0.167
**Template (6)**	**0.630±0.033**	3.652±0.182	**0.514±0.033**	3.358±0.172

Mean and standard deviation of the performance across 100 repetitions and cross-validations are reported. Performance was evaluated using accuracy for classification and mean absolute error for regression. The top three performances are reported in bold.

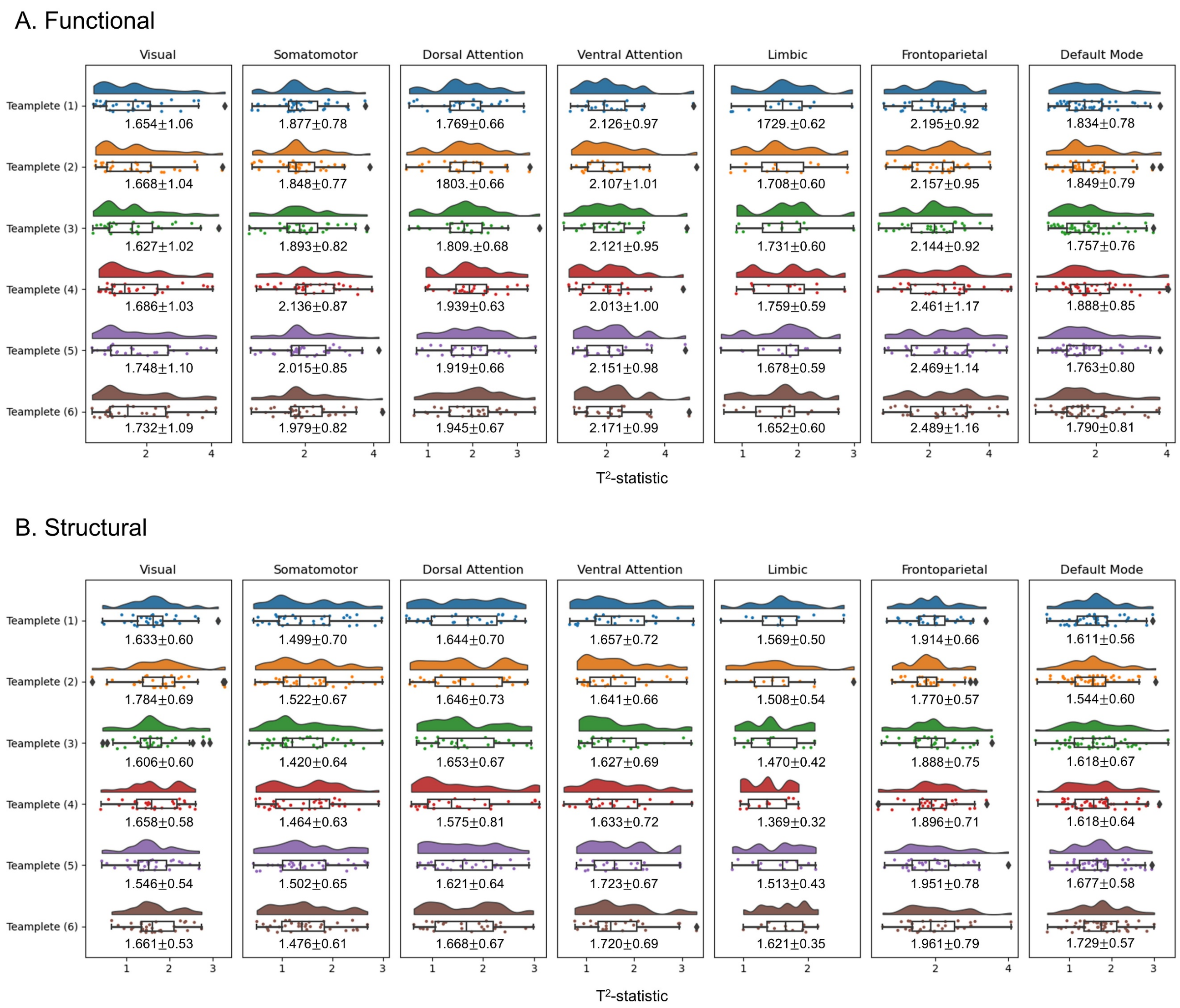

Supplementary Fig. 1 | Between-group differences in the first three connectivity gradients between individuals with ASD and neurotypical controls based on different group-level templates quantified over functional communities. **(A)** We stratified the between-group difference effects (Hotelling's T^2) using functional and **(B)** structural gradients according to seven functional communities using rain cloud plots, with mean and standard deviation.

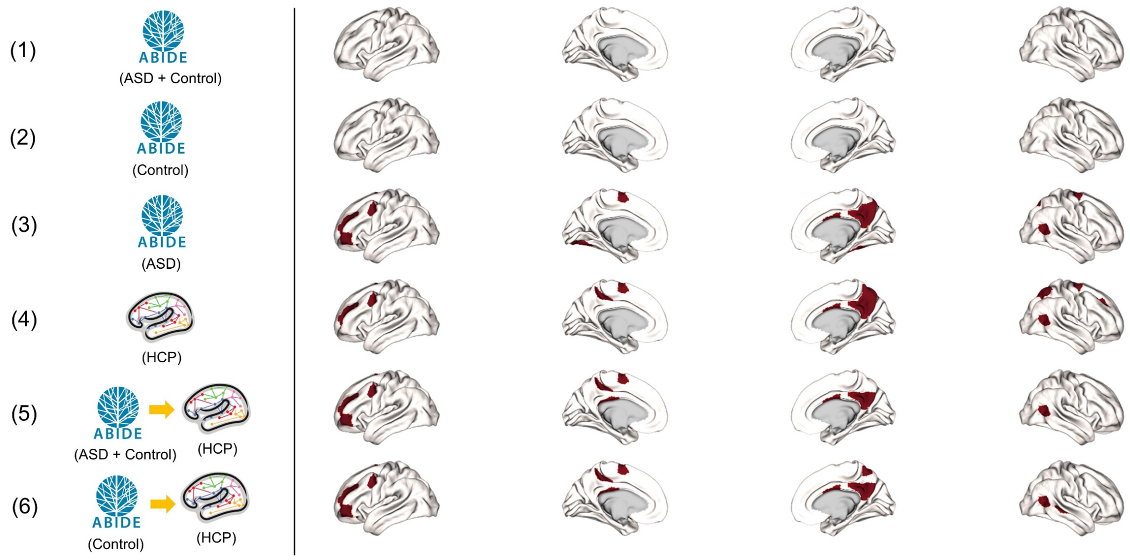

Supplementary Fig. 2 | Between-group differences in structural connectivity gradients between individuals with autism spectrum disorder and neurotypical controls according to different template gradients. The regions showed moderate ($p_{FDR} < 0.1$) between-group differences in three gradients marked in red. Abbreviations: ASD, autism spectrum disorder; HCP, Human Connectome Project; ABIDE, Autism Brain Imaging Data Exchange Initiative.

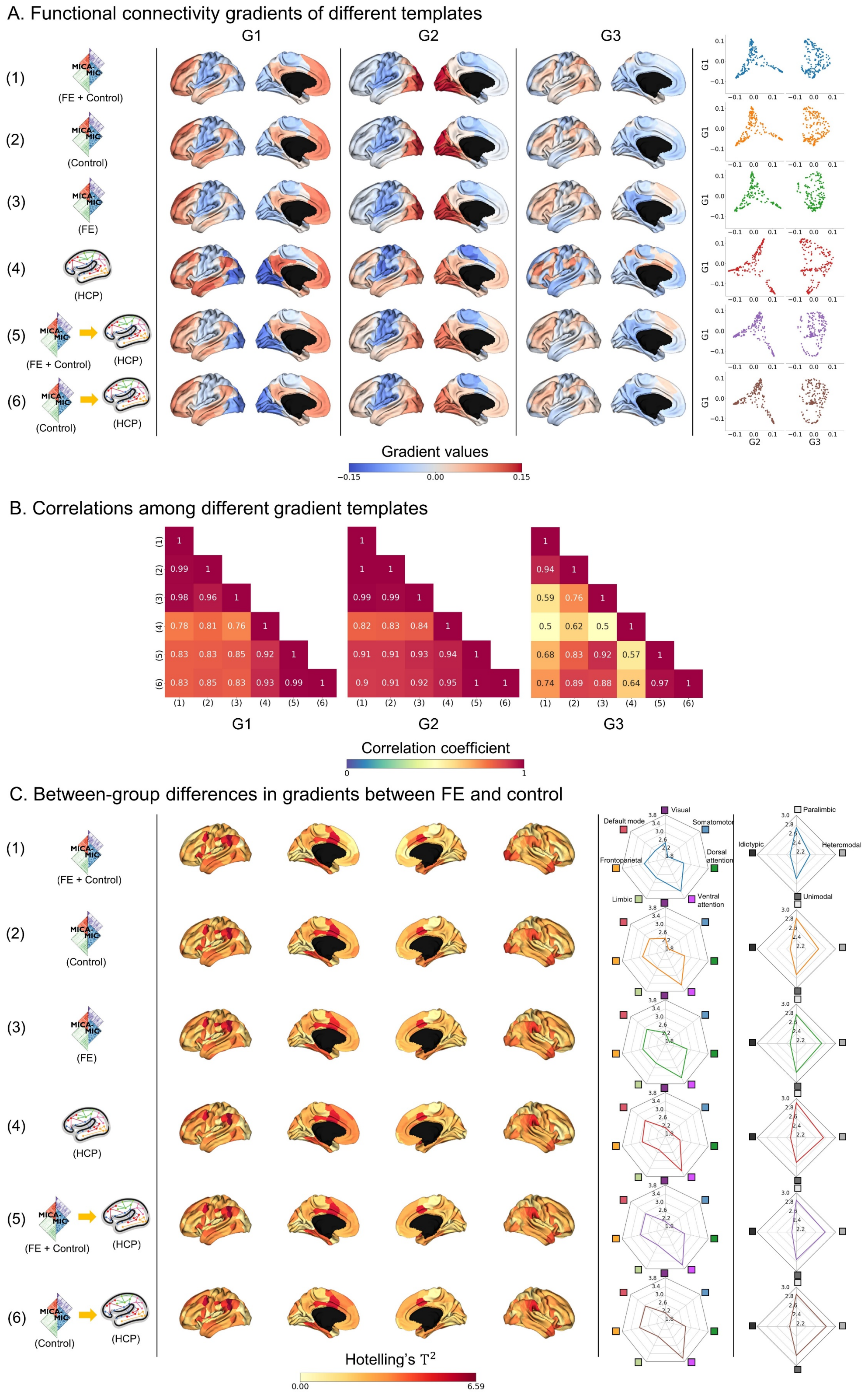

Supplementary Fig. 3 | Between-group differences in functional connectivity gradients between individuals with FE and healthy controls according to different template gradients. **(A)** Three functional connectivity gradients (G1, G2, and G3) generated by six different template strategies were plotted on brain surfaces, and the scatter plots displayed the distribution of gradient pairs. **(B)** A heatmap of the correlation coefficients among different template pairs was reported. **(C)** Hotelling's T^2 statistics of the whole brain were plotted on brain surfaces and stratified according to seven functional communities and four cortical hierarchical levels using radar plots. Abbreviations: ASD, autism spectrum disorder; HCP, Human Connectome Project; FE, focal epilepsy.

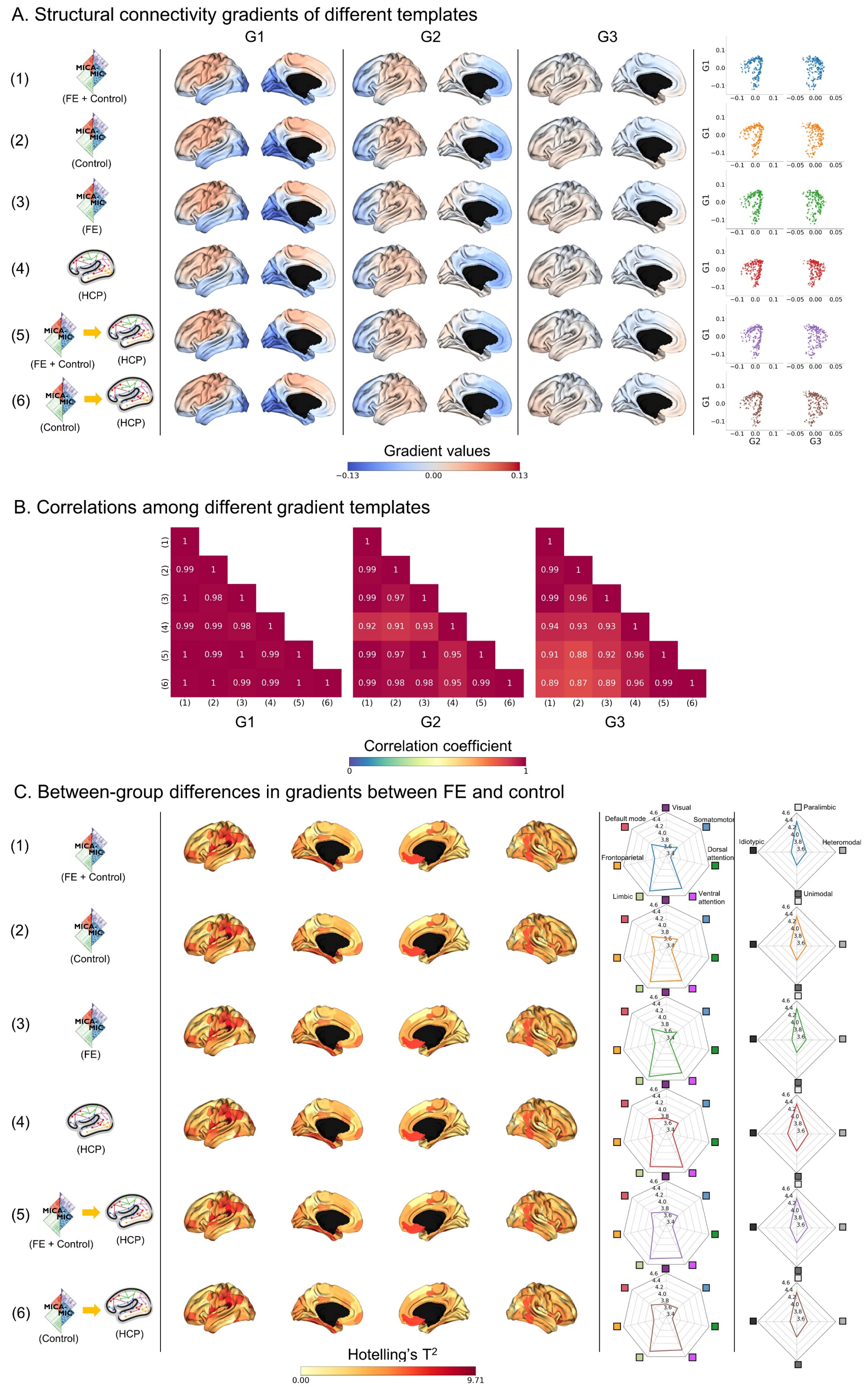

Supplementary Fig. 4 | Between-group differences in structural connectivity gradients between individuals with FE and healthy controls according to different template gradients. **(A)** Three structural connectivity gradients (G1, G2, and G3) generated by six different template strategies were plotted on brain surfaces, and the scatter plots displayed the distribution of gradient pairs. **(B)** A heatmap of the correlation coefficients among different template pairs was reported. **(C)** Hotelling's T^2 statistics of the whole brain were plotted on brain surfaces and stratified according to seven functional communities and four cortical hierarchical levels using radar plots. Abbreviations: ASD, autism spectrum disorder; HCP, Human Connectome Project; FE, focal epilepsy.

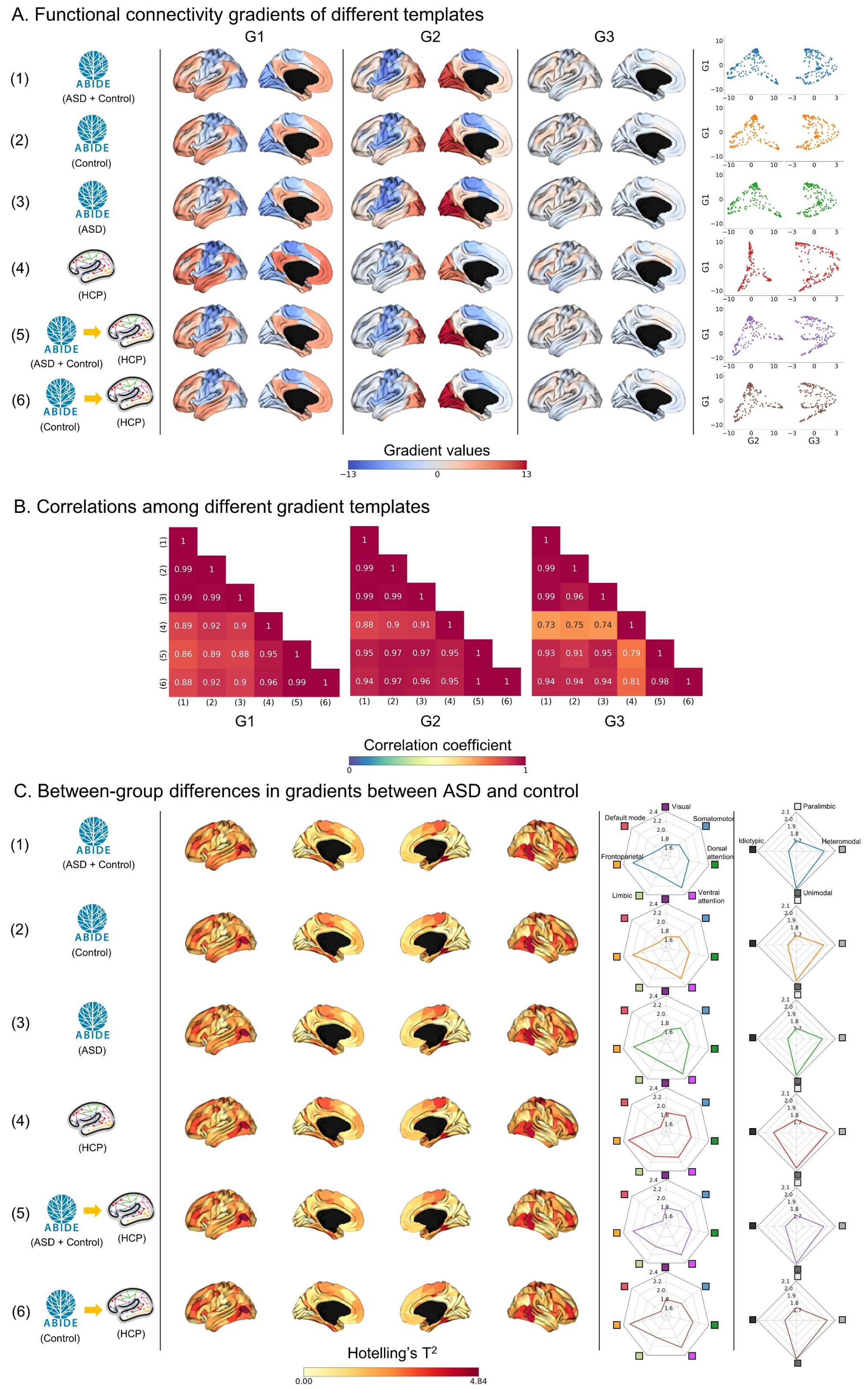

Supplementary Fig. 5 | Functional connectivity gradients of different templates as well as between-group differences performed using the cosine similarity kernel. **(A)** Three functional connectivity gradients (G1, G2, and G3) generated by six different template strategies were plotted on brain surfaces, and the scatter plots showed the distribution of gradient pairs. **(B)** A heatmap of the correlation coefficients among different template pairs was reported. **(C)** Hotelling's T^2 statistics of the whole brain were plotted on brain surfaces and stratified according to seven functional communities and four cortical hierarchical levels using radar plots. Abbreviations: ASD, autism spectrum disorder; HCP, Human Connectome Project; ABIDE, Autism Brain Imaging Data Exchange Initiative.

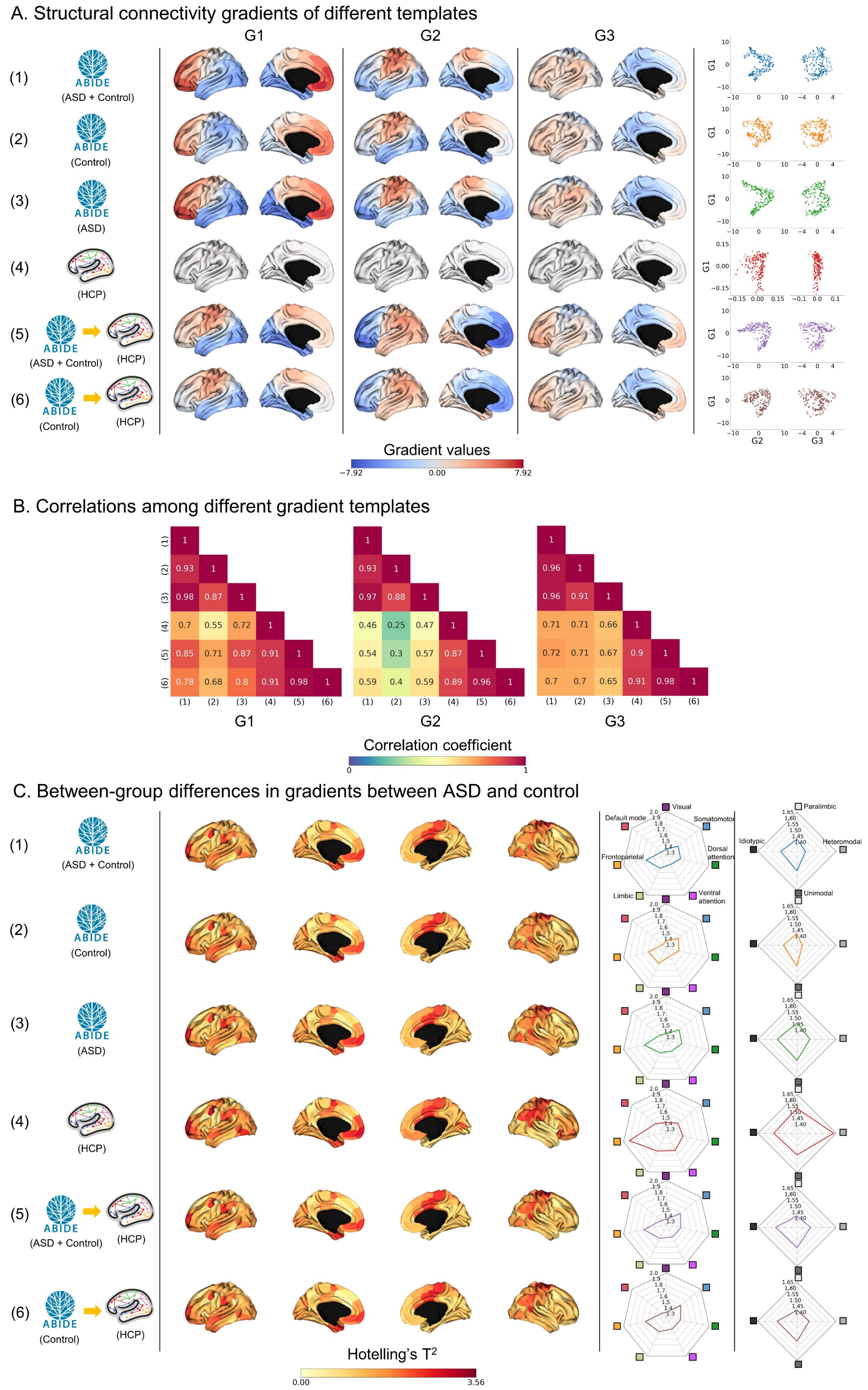

Supplementary Fig. 6 | Structural connectivity gradients of different templates as well as between-group differences performed using the cosine similarity kernel. **(A)** Three structural connectivity gradients (G1, G2, and G3) generated by six different template strategies were plotted on brain surfaces, and the scatter plots showed the distribution of gradient pairs. **(B)** A heatmap of the correlation coefficients among different template pairs was reported. **(C)** Hotelling's T^2 statistics of the whole brain were plotted on brain surfaces and stratified according to seven functional communities and four cortical hierarchical levels using radar plots. Abbreviations: ASD, autism spectrum disorder; HCP, Human Connectome Project; ABIDE, Autism Brain Imaging Data Exchange Initiative.

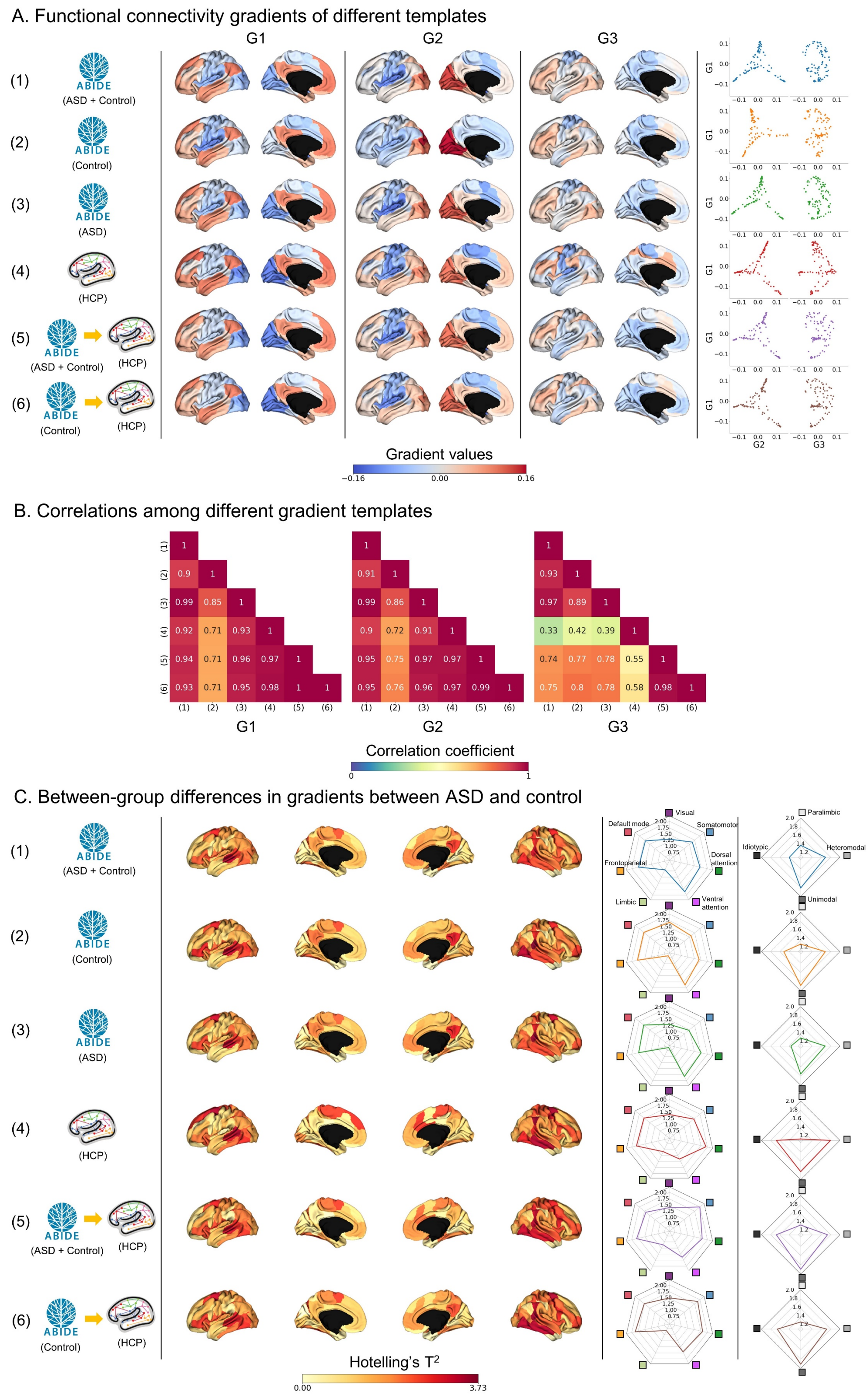

Supplementary Fig. 7 | Functional connectivity gradients of different templates as well as between-group differences performed using Schaefer 100 parcellation. **(A)** Three functional connectivity gradients (G1, G2, and G3) generated by six different template strategies were plotted on brain surfaces, and the scatter plots showed the distribution of gradient pairs. **(B)** A heatmap of the correlation coefficients among different template pairs was reported. **(C)** Hotelling's T^2 statistics of the whole brain were plotted on brain surfaces and stratified according to seven functional communities and four cortical hierarchical levels using radar plots. Abbreviations: ASD, autism spectrum disorder; HCP, Human Connectome Project; ABIDE, Autism Brain Imaging Data Exchange Initiative.

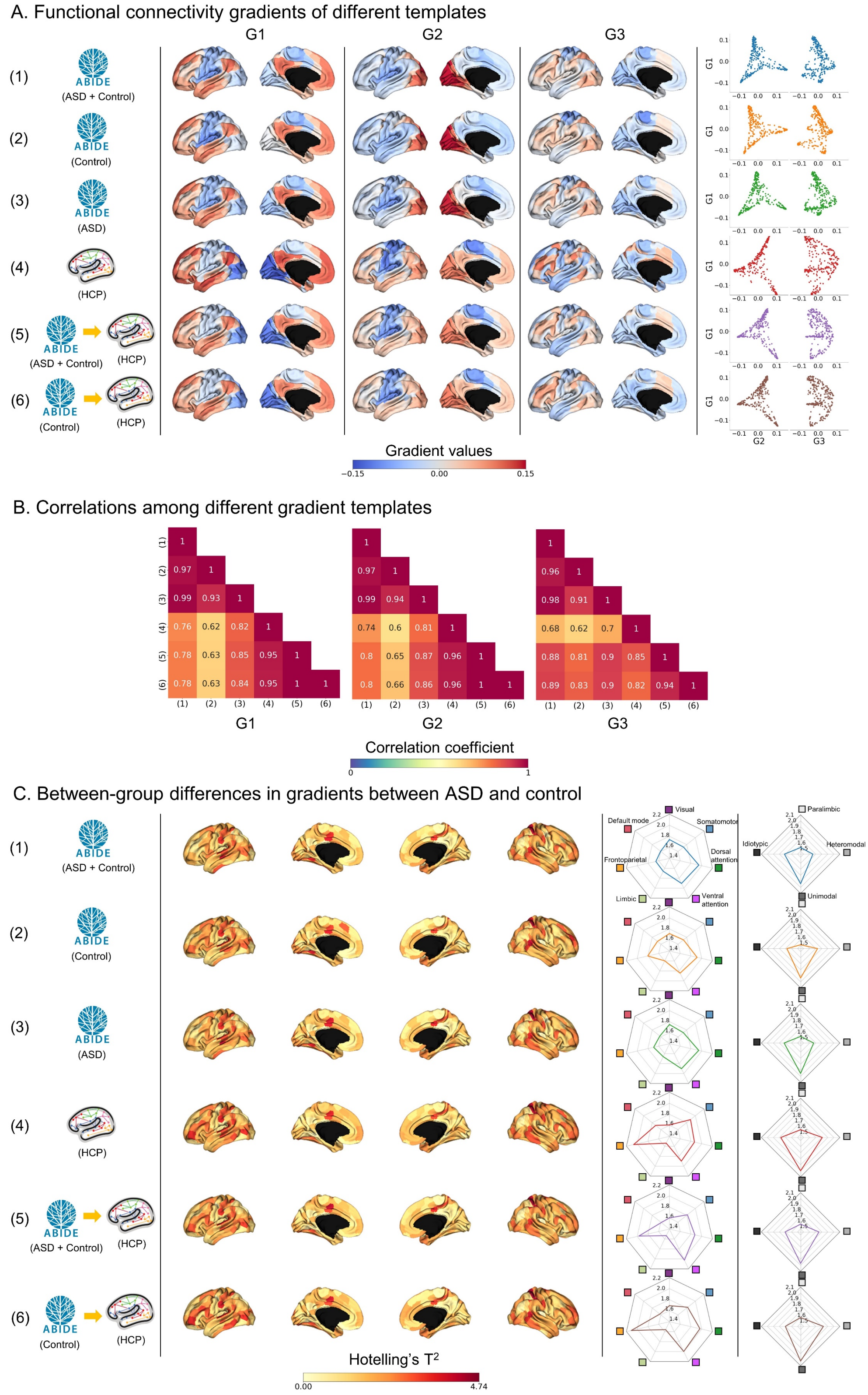

Supplementary Fig. 8 | Functional connectivity gradients of different templates as well as between-group differences performed using Schaefer 300 parcellation. **(A)** Three functional connectivity gradients (G1, G2, and G3) generated by six different template strategies were plotted on brain surfaces, and the scatter plots showed the distribution of gradient pairs. **(B)** A heatmap of the correlation coefficients among different template pairs was reported. **(C)** Hotelling's T^2 statistics of the whole brain were plotted on brain surfaces and stratified according to seven functional communities and four cortical hierarchical levels using radar plots. Abbreviations: ASD, autism spectrum disorder; HCP, Human Connectome Project; ABIDE, Autism Brain Imaging Data Exchange Initiative.

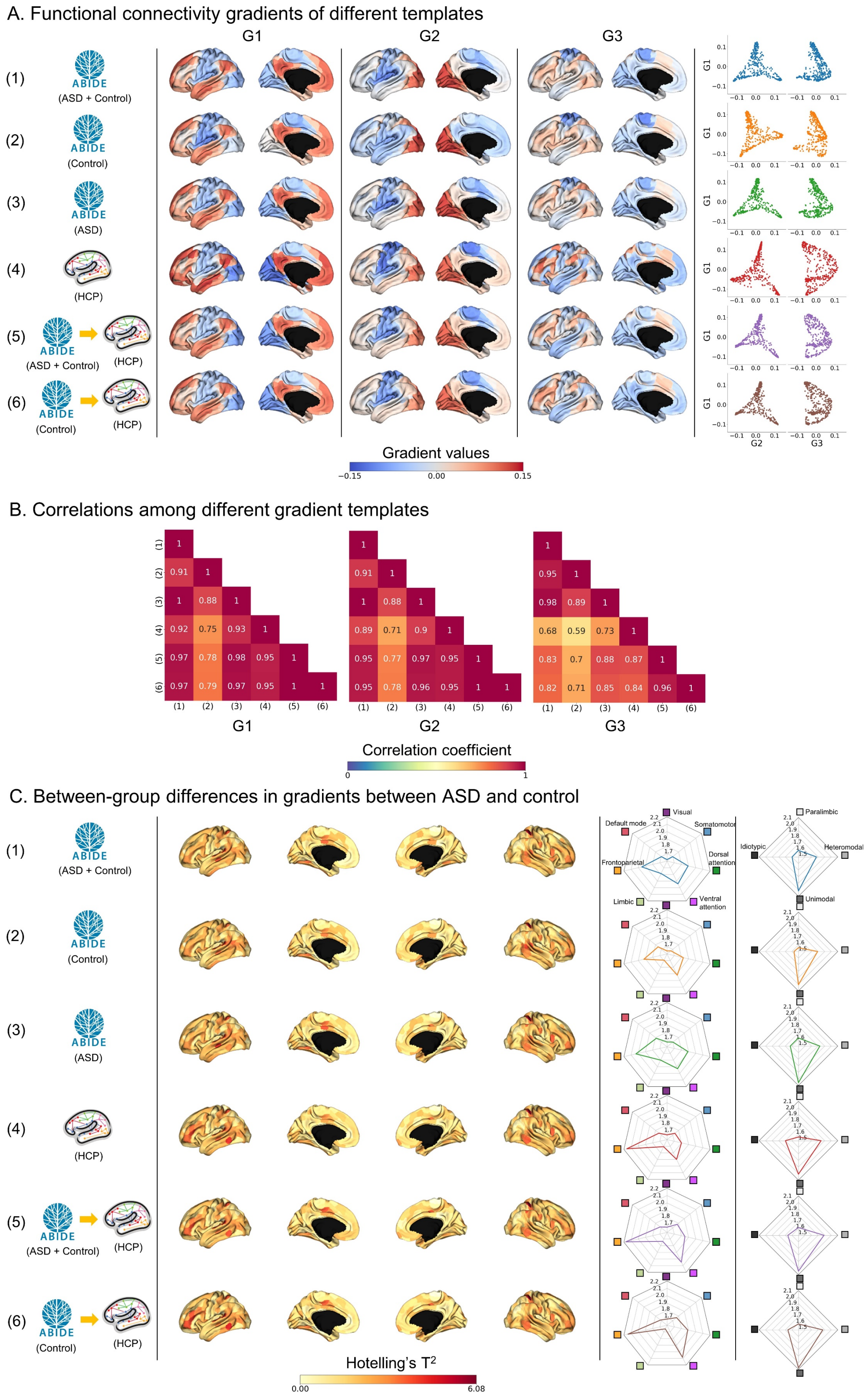

Supplementary Fig. 9 | Functional gradients of different templates and between-group differences using 400 parcels. (A) We generated three functional connectivity gradients (G1, G2, and G3) of six different template strategies and plotted them on brain surfaces. The scatter plots display the distribution of gradient pairs. **(B)** We reported heatmaps of correlation coefficients between different template pairs. **(C)** Hotelling's T^2 statistics of the whole brain are plotted on brain surfaces. We stratified the effects according to seven functional communities and four cortical hierarchical levels using radar plots. Abbreviations: ASD, autism spectrum disorder; HCP, Human Connectome Project; ABIDE, Autism Brain Imaging Data Exchange Initiative.

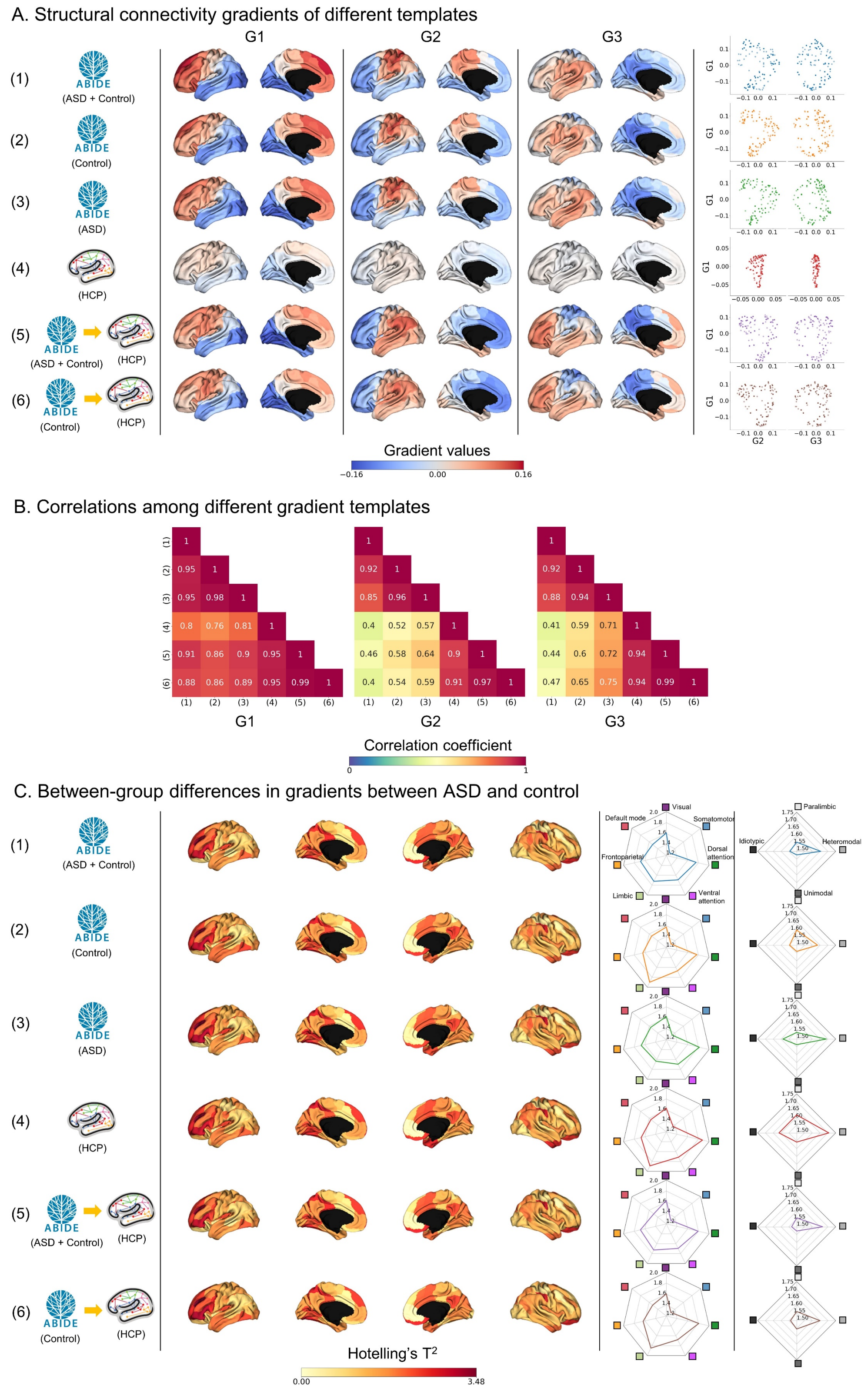

Supplementary Fig. 10 | Structural connectivity gradients of different templates as well as between-group differences performed using Schaefer 100 parcellation. **(A)** Three structural connectivity gradients (G1, G2, and G3) generated by six different template strategies were plotted on brain surfaces, and the scatter plots showed the distribution of gradient pairs. **(B)** A heatmap of the correlation coefficients among different template pairs was reported. **(C)** Hotelling's T^2 statistics of the whole brain were plotted on brain surfaces and stratified according to seven functional communities and four cortical hierarchical levels using radar plots. Abbreviations: ASD, autism spectrum disorder; HCP, Human Connectome Project; ABIDE, Autism Brain Imaging Data Exchange Initiative.

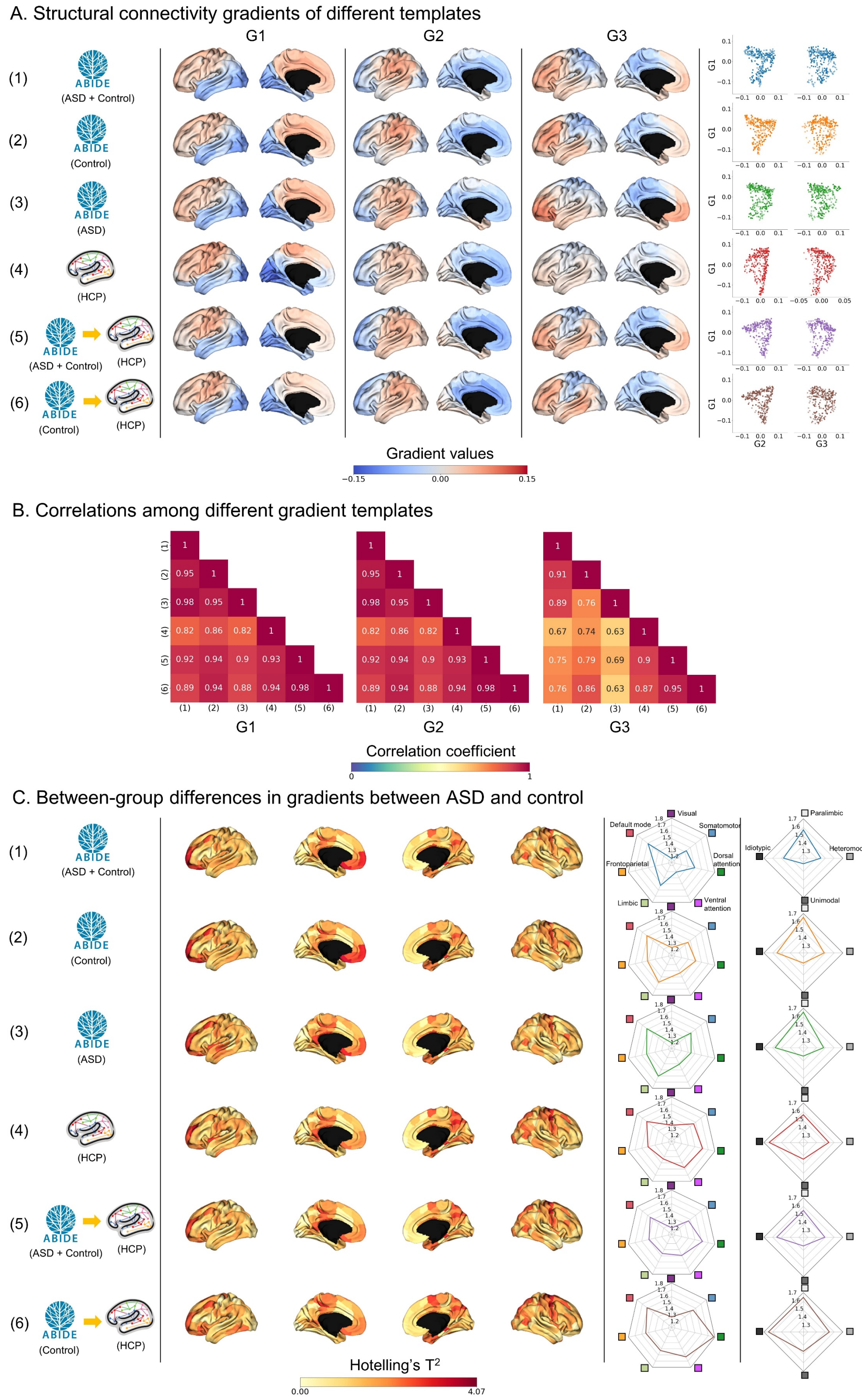

Supplementary Fig. 11 | Structural connectivity gradients of different templates as well as between-group differences performed using Schaefer 300 parcellation. **(A)** Three structural connectivity gradients (G1, G2, and G3) generated by six different template strategies were plotted on brain surfaces, and the scatter plots showed the distribution of gradient pairs. **(B)** A heatmap of the correlation coefficients among different template pairs was reported. **(C)** Hotelling's T^2 statistics of the whole brain were plotted on brain surfaces and stratified according to seven functional communities and four cortical hierarchical levels using radar plots. Abbreviations: ASD, autism spectrum disorder; HCP, Human Connectome Project; ABIDE, Autism Brain Imaging Data Exchange Initiative.

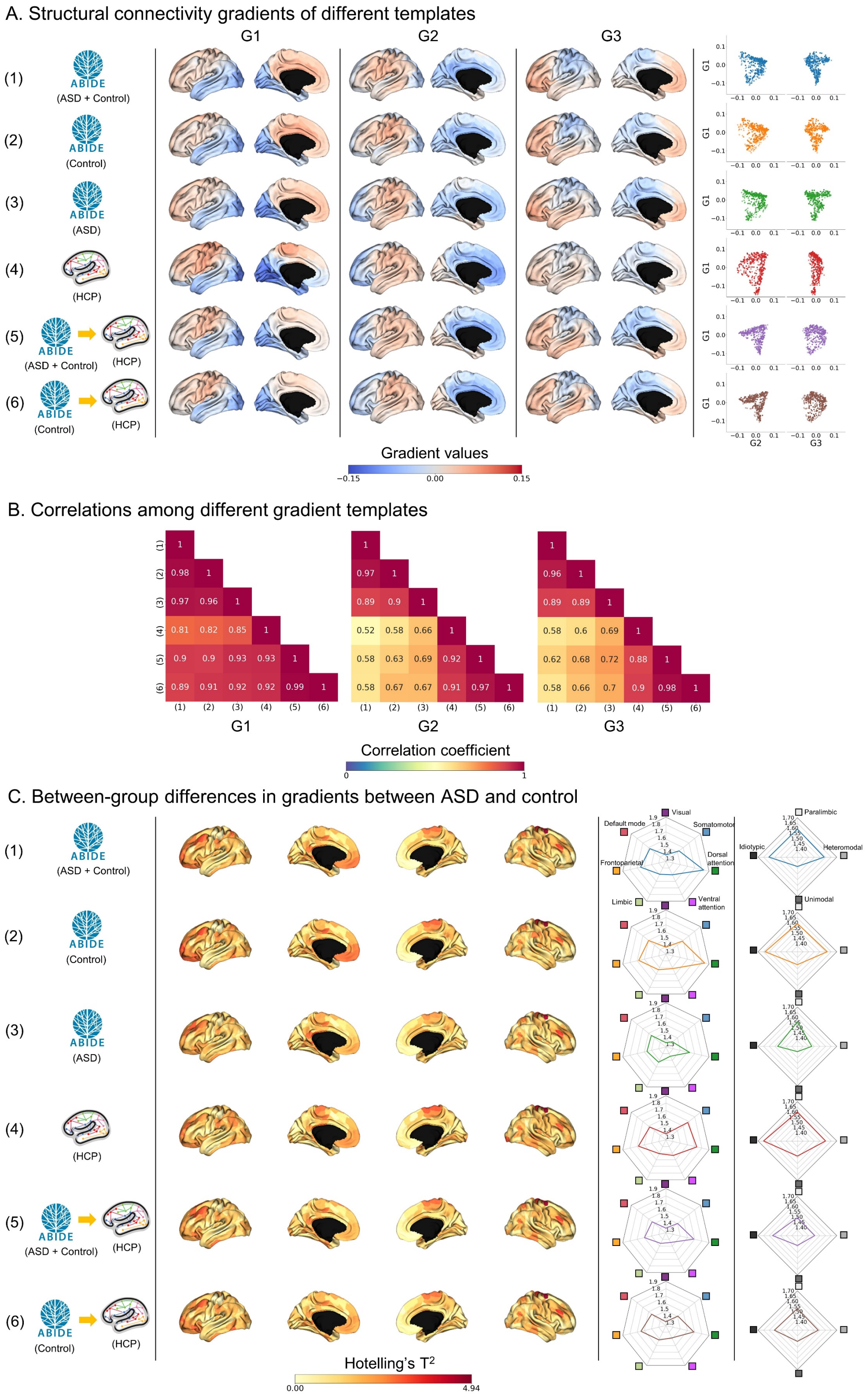

Supplementary Fig. 12 | Structural gradients of different templates and between-group differences using 400 parcels. **(A)** We generated three structural connectivity gradients (G1, G2, and G3) of six different template strategies and plotted them on brain surfaces. The scatter plots display the distribution of gradient pairs. **(B)** We reported heatmaps of correlation coefficients between different template pairs. **(C)** Hotelling's T^2 statistics of the whole brain are plotted on brain surfaces. We stratified the effects according to seven functional communities and four cortical hierarchical levels using radar plots. Abbreviations: ASD, autism spectrum disorder; HCP, Human Connectome Project; ABIDE, Autism Brain Imaging Data Exchange Initiative.

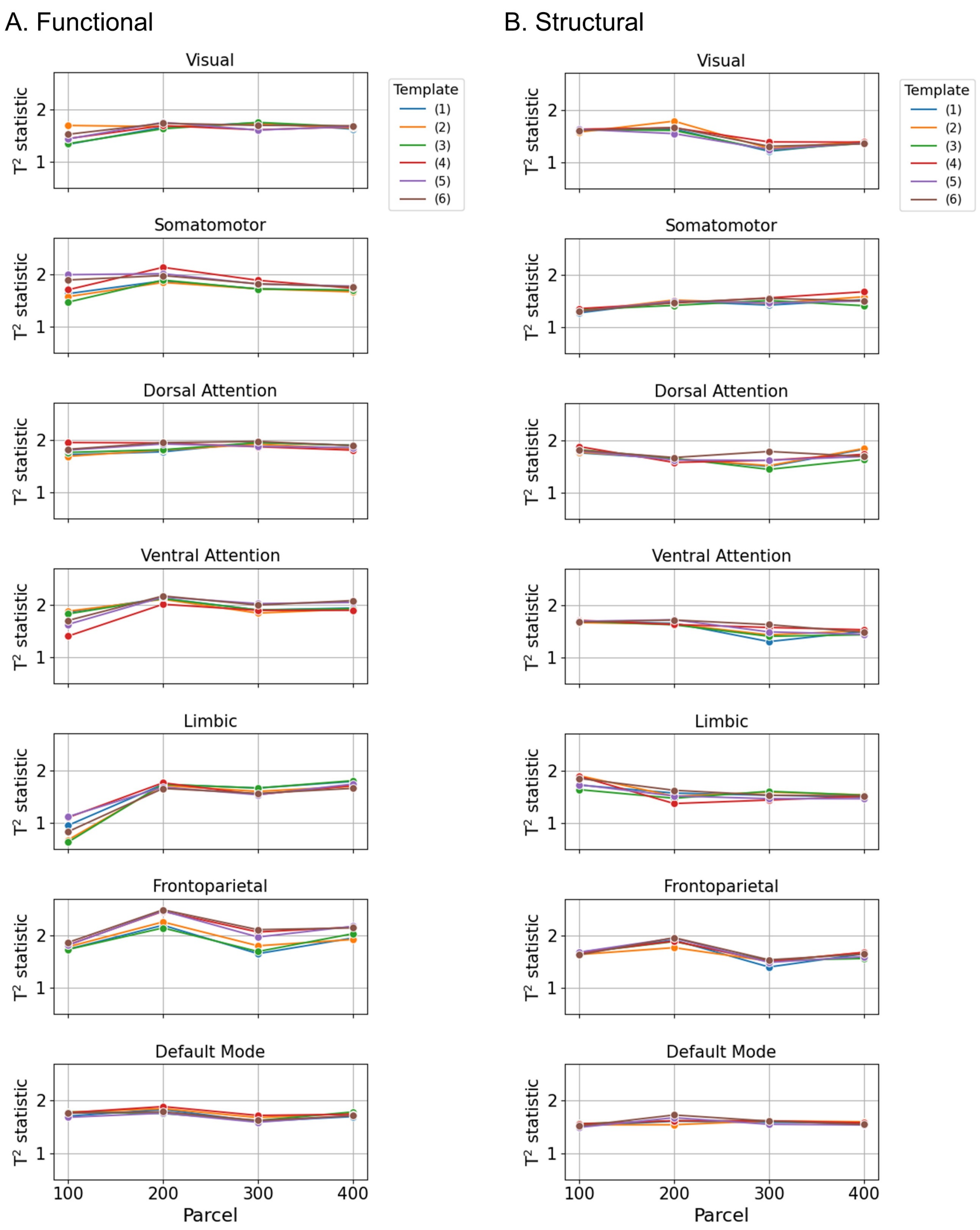

Supplementary Fig. 13 | Comparative analysis across various spatial granularities of the Schafer atlas. We plotted the trajectories of the between-group difference effect sizes between the ASD and control groups using **(A)** functional and **(B)** structural gradients across different parcel sizes.

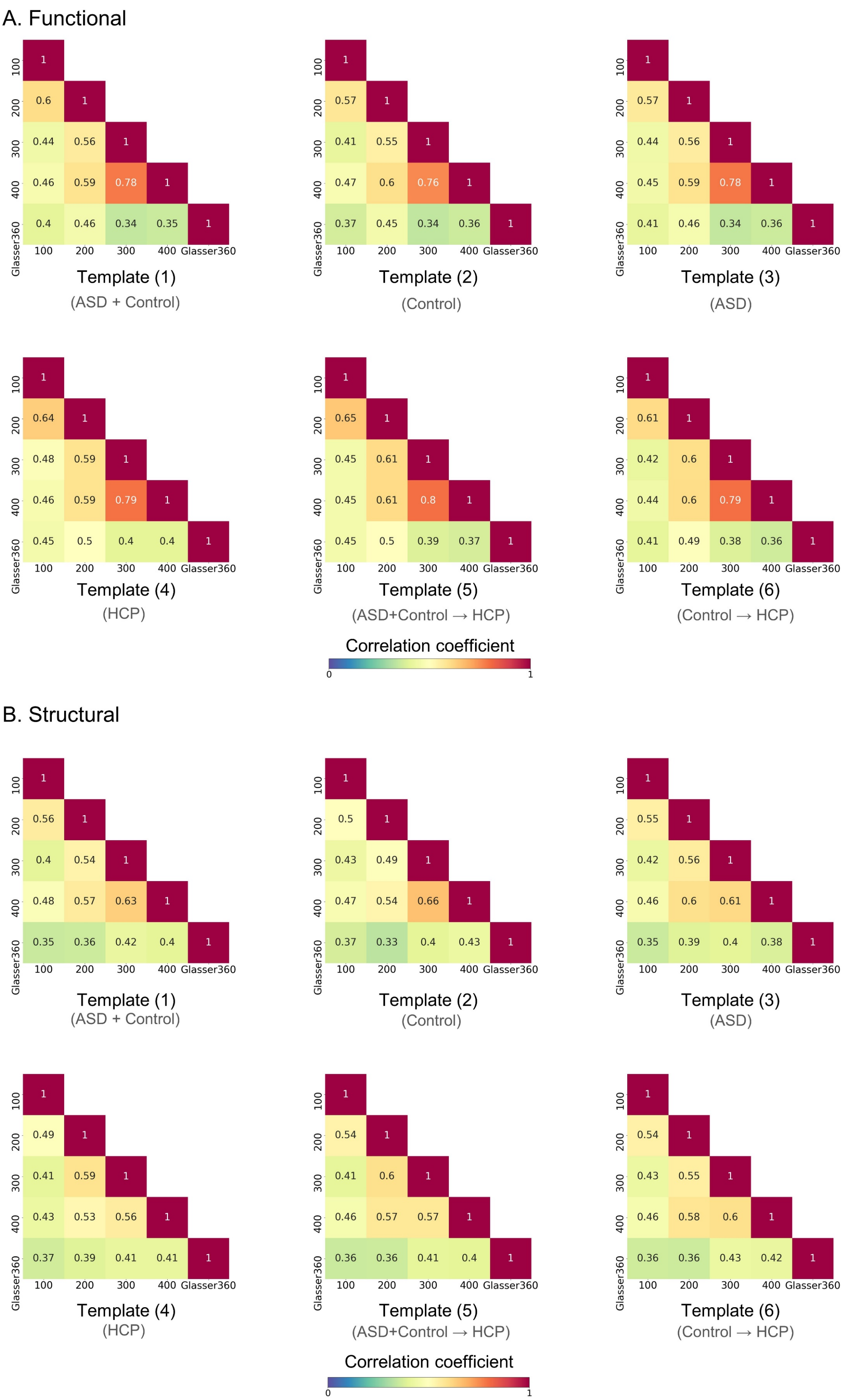

Supplementary Fig. 14 | Quantitative analysis across different atlases. The heatmaps of spatial correlation coefficients in Hotelling's T^2 statistic map using **(A)** functional and **(B)** structural gradients across different atlases are plotted. We compared Schafer atlas with 100, 200, 300, and 400 parcels besides the Glasser360 atlas.

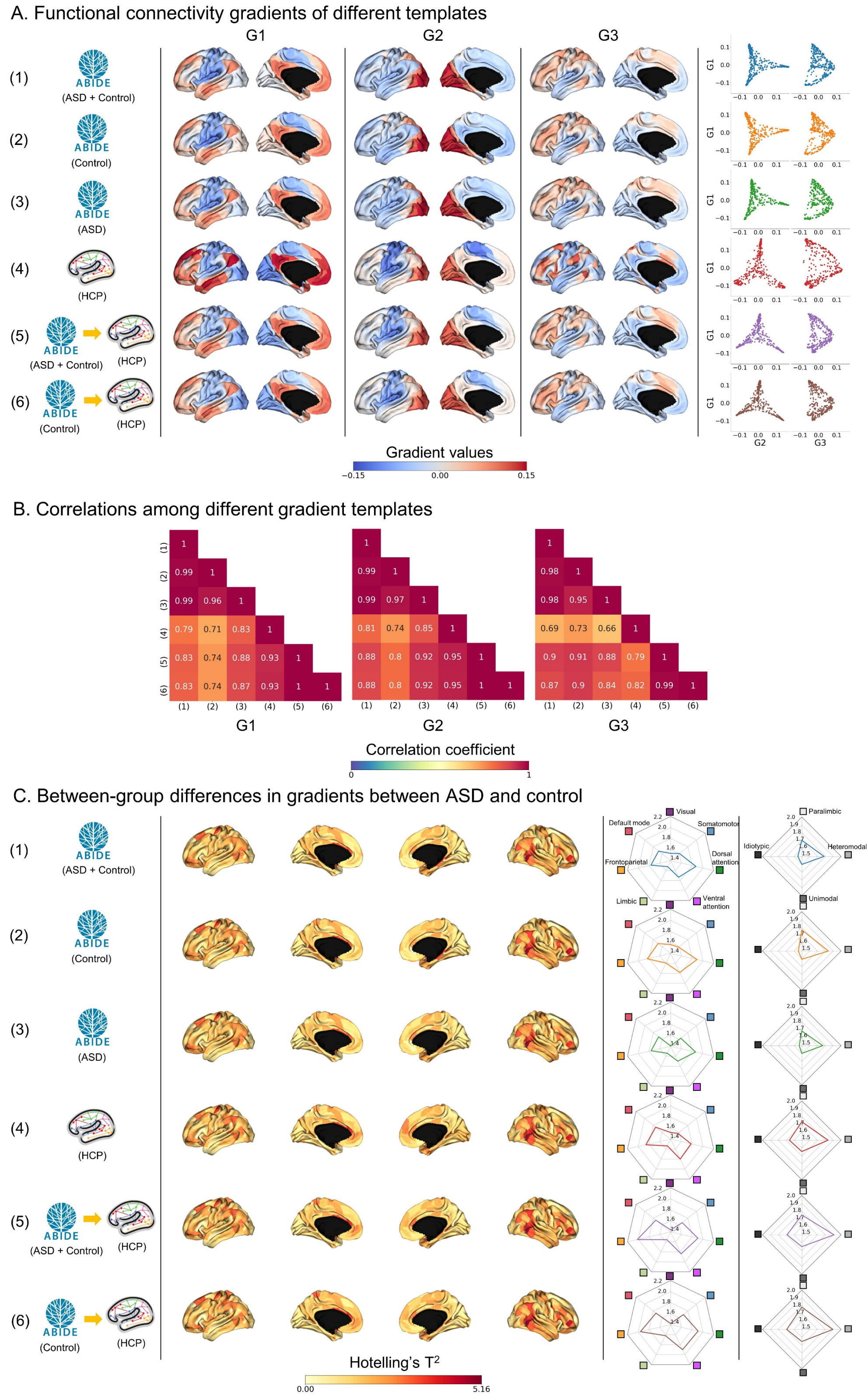

Supplementary Fig. 15 | Functional gradients of different templates and between-group differences using the Glasser360 atlas. (A) We generated three functional connectivity gradients (G1, G2, and G3) of six different template strategies and plotted them on brain surfaces. The scatter plots display the distribution of gradient pairs. **(B)** We reported heatmaps of correlation coefficients between different template pairs. **(C)** Hotelling's T^2 statistics of the whole brain are plotted on brain surfaces. We stratified the effects according to seven functional communities and four cortical hierarchical levels using radar plots. Abbreviations: ASD, autism spectrum disorder; HCP, Human Connectome Project; ABIDE, Autism Brain Imaging Data Exchange Initiative.

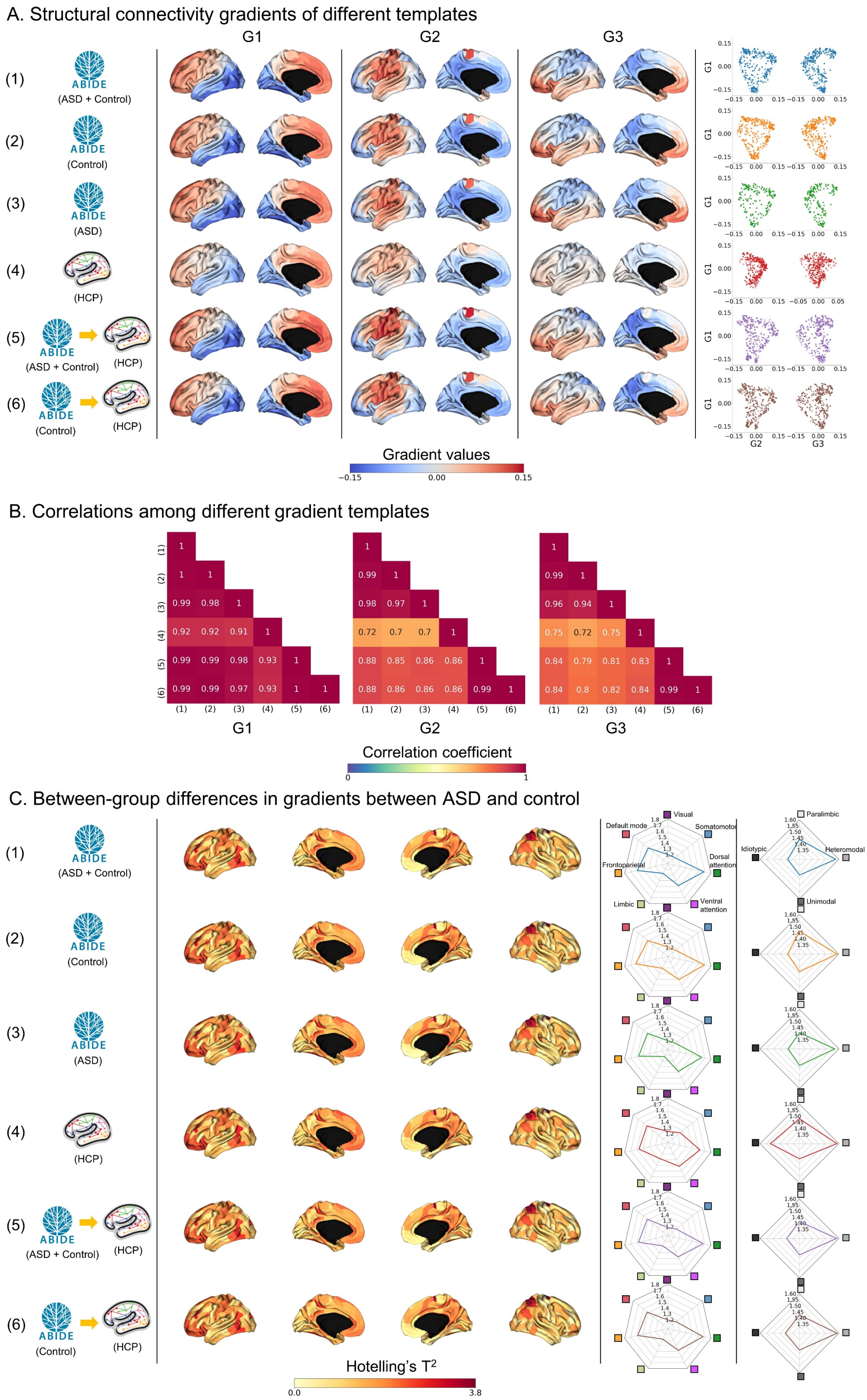

Supplementary Fig. 16 | Structural gradients of different templates and between-group differences using Glasser360 atlas. (A) We generated three structural connectivity gradients (G1, G2, and G3) of six different template strategies and plotted them on brain surfaces. The scatter plots display the distribution of gradient pairs. **(B)** We reported heatmaps of correlation coefficients between different template pairs. **(C)** Hotelling's T^2 statistics of the whole brain are plotted on brain surfaces. We stratified the effects according to seven functional communities and four cortical hierarchical levels using radar plots. Abbreviations: ASD, autism spectrum disorder; HCP, Human Connectome Project; ABIDE, Autism Brain Imaging Data Exchange Initiative.

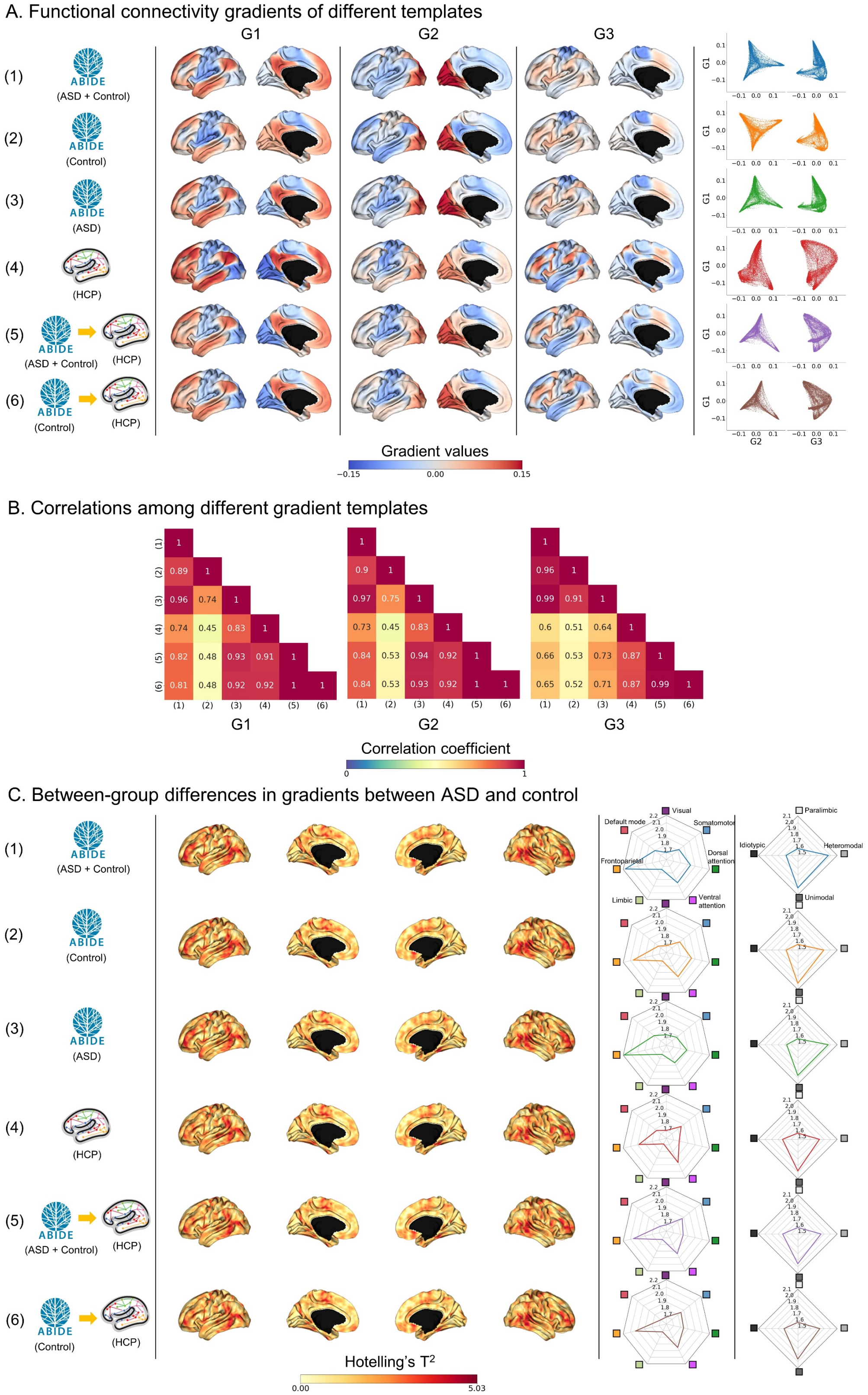

Supplementary Fig. 17 | Vertex-wise functional gradients of different templates and between-group differences. (A) We generated three functional connectivity gradients (G1, G2, and G3) of six different template strategies and plotted them on brain surfaces. The scatter plots display the distribution of gradient pairs. **(B)** We reported heatmaps of correlation coefficients between different template pairs. **(C)** Hotelling's T^2 statistics of the whole brain are plotted on brain surfaces. We stratified the effects according to seven functional communities and four cortical hierarchical levels using radar plots. Abbreviations: ASD, autism spectrum disorder; HCP, Human Connectome Project; ABIDE, Autism Brain Imaging Data Exchange Initiative.

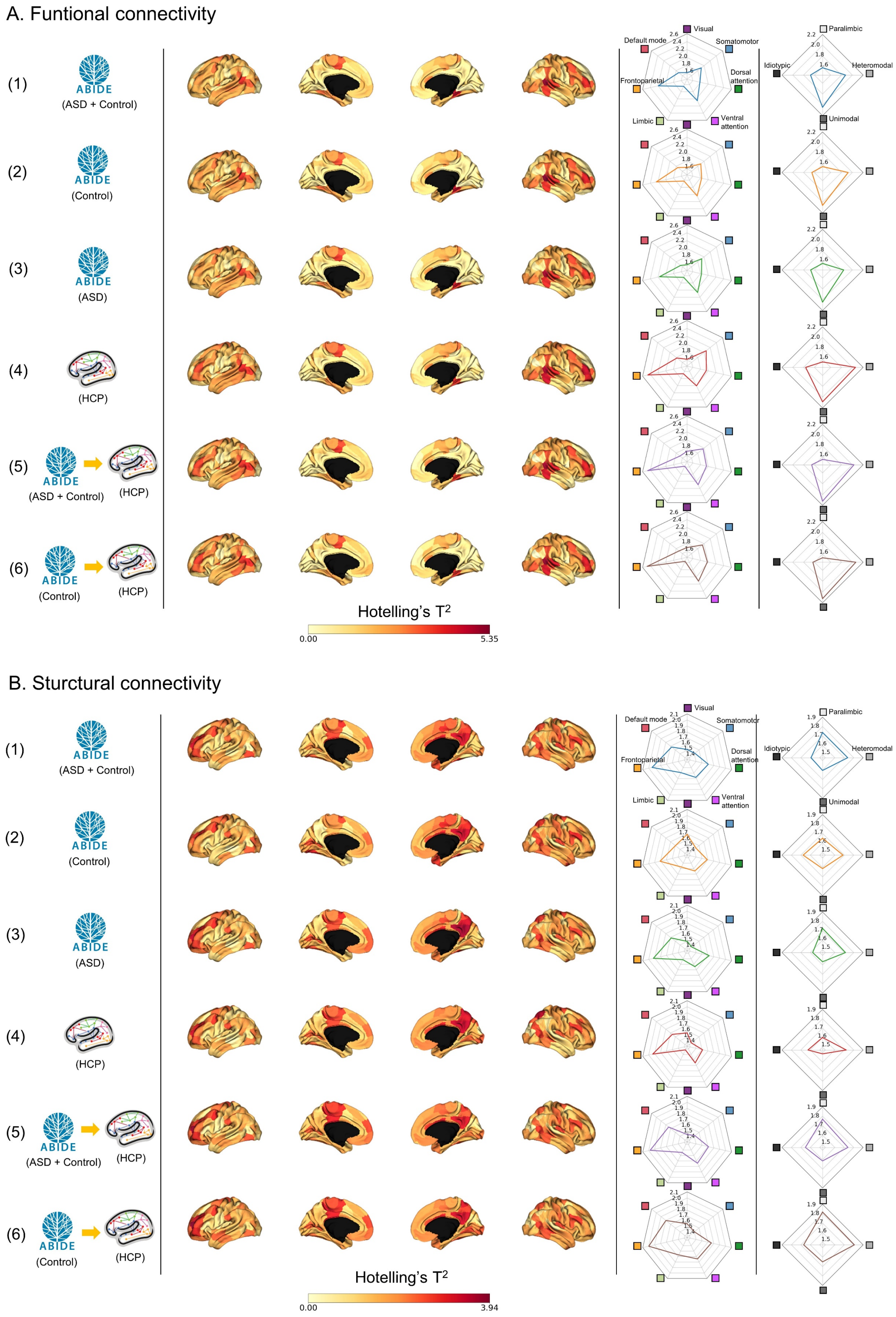

Supplementary Fig. 18 | Between-group differences in gradients between ASD and control with additional head motion control. **(A)** Three functional and **(B)** structural connectivity gradients (G1, G2, and G3) generated by six different template strategies were used. Hotelling's T^2 statistics of the whole brain were plotted on brain surfaces and stratified according to seven functional communities and four cortical hierarchical levels using radar plots. Abbreviations: ASD, autism spectrum disorder; HCP, Human Connectome Project; ABIDE, Autism Brain Imaging Data Exchange Initiative.

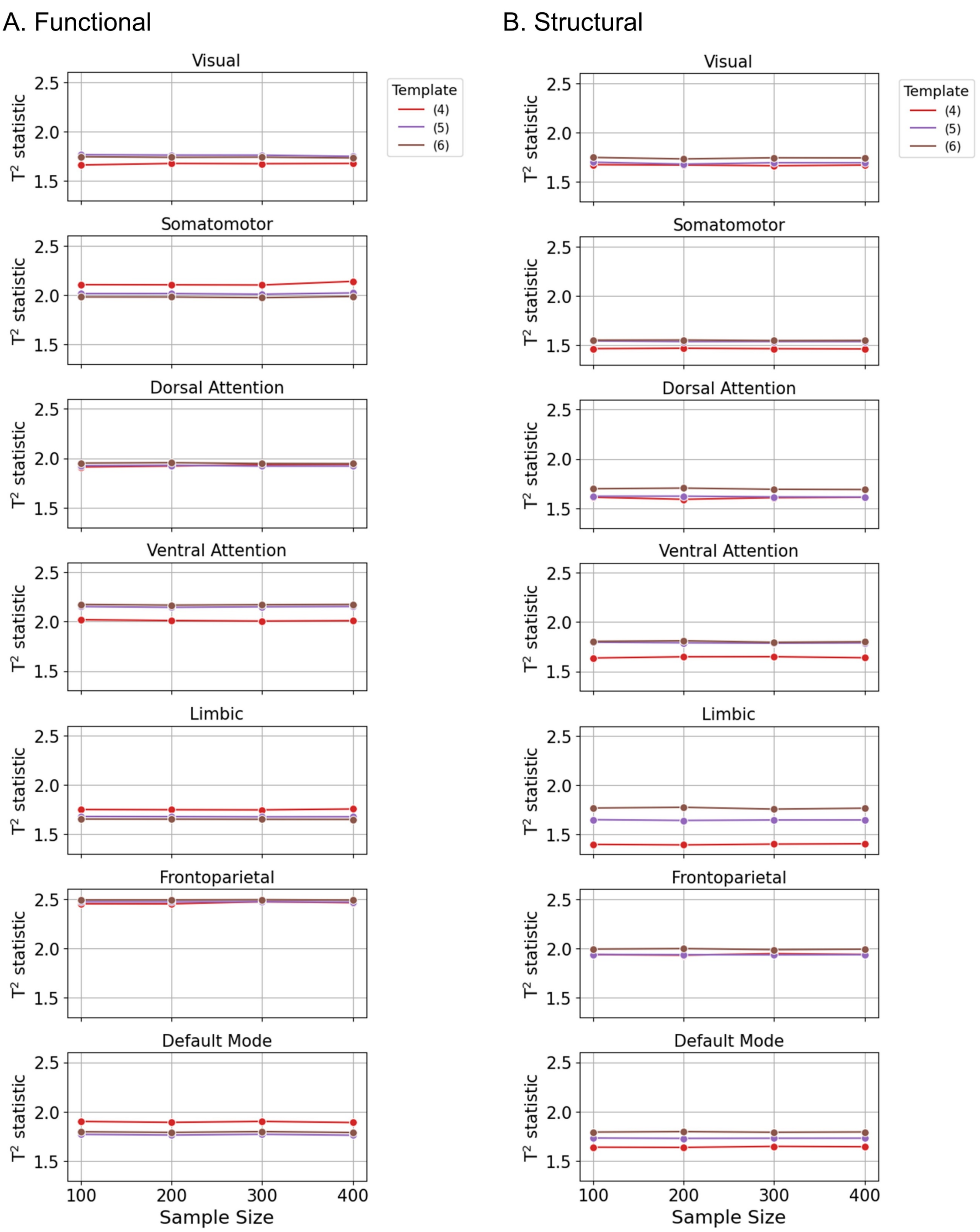

Supplementary Fig. 19 | Between-group differences using group-level templates constructed using subsets of HCP subjects. (A) The effect sizes using functional and **(B)** structural gradients across different group-level templates with varying sample sizes are stratified according to seven functional communities.

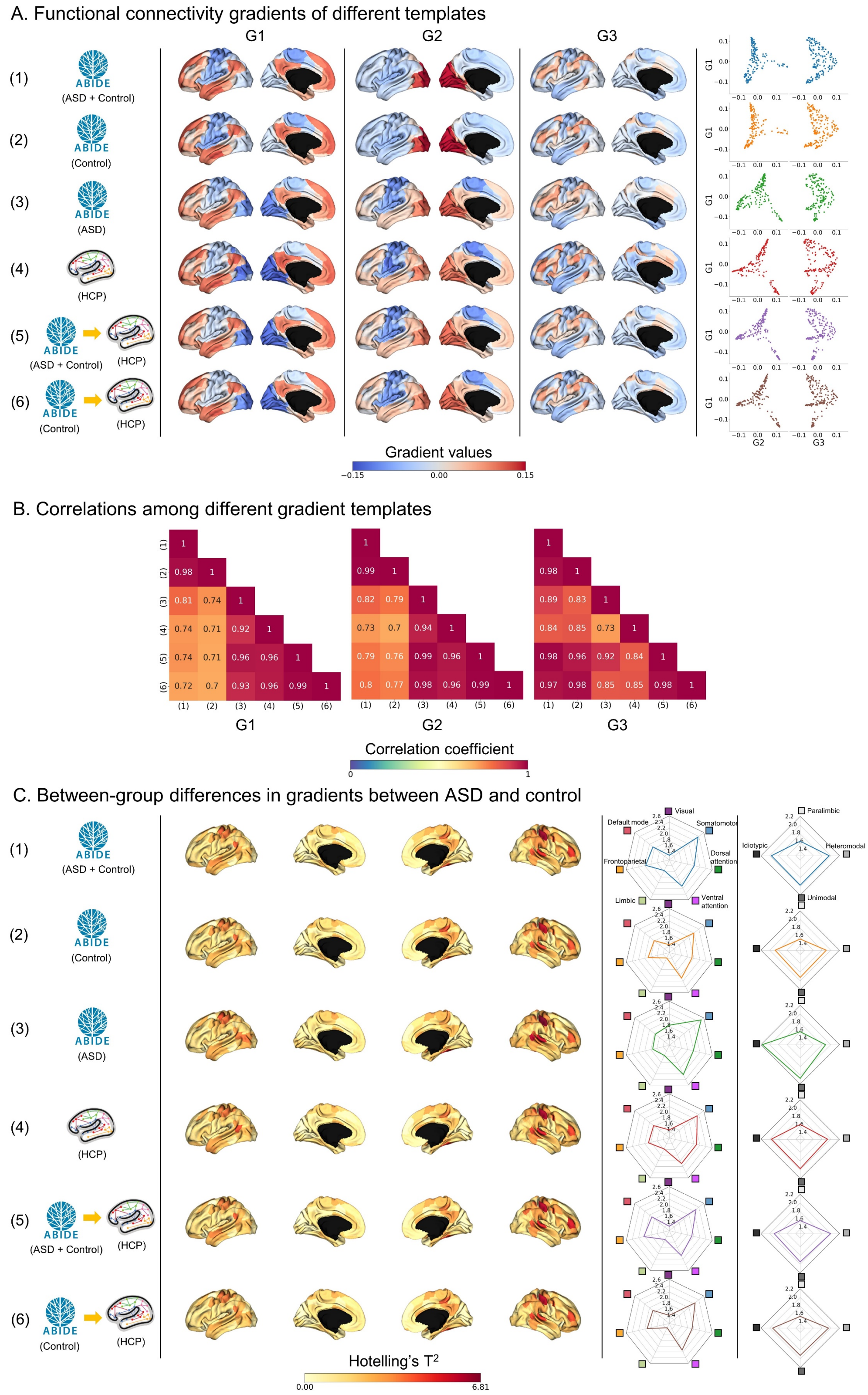

Supplementary Fig. 20 | Functional gradients of different templates and between-group differences in IP site. (A) We generated three functional connectivity gradients (G1, G2, and G3) of six different template strategies and plotted them on brain surfaces. The scatter plots display the distribution of gradient pairs. **(B)** We reported heatmaps of correlation coefficients between different template pairs. **(C)** Hotelling's T^2 statistics of the whole brain are plotted on brain surfaces. We stratified the effects according to seven functional communities and four cortical hierarchical levels using radar plots. Abbreviations: ASD, autism spectrum disorder; HCP, Human Connectome Project; ABIDE, Autism Brain Imaging Data Exchange Initiative.

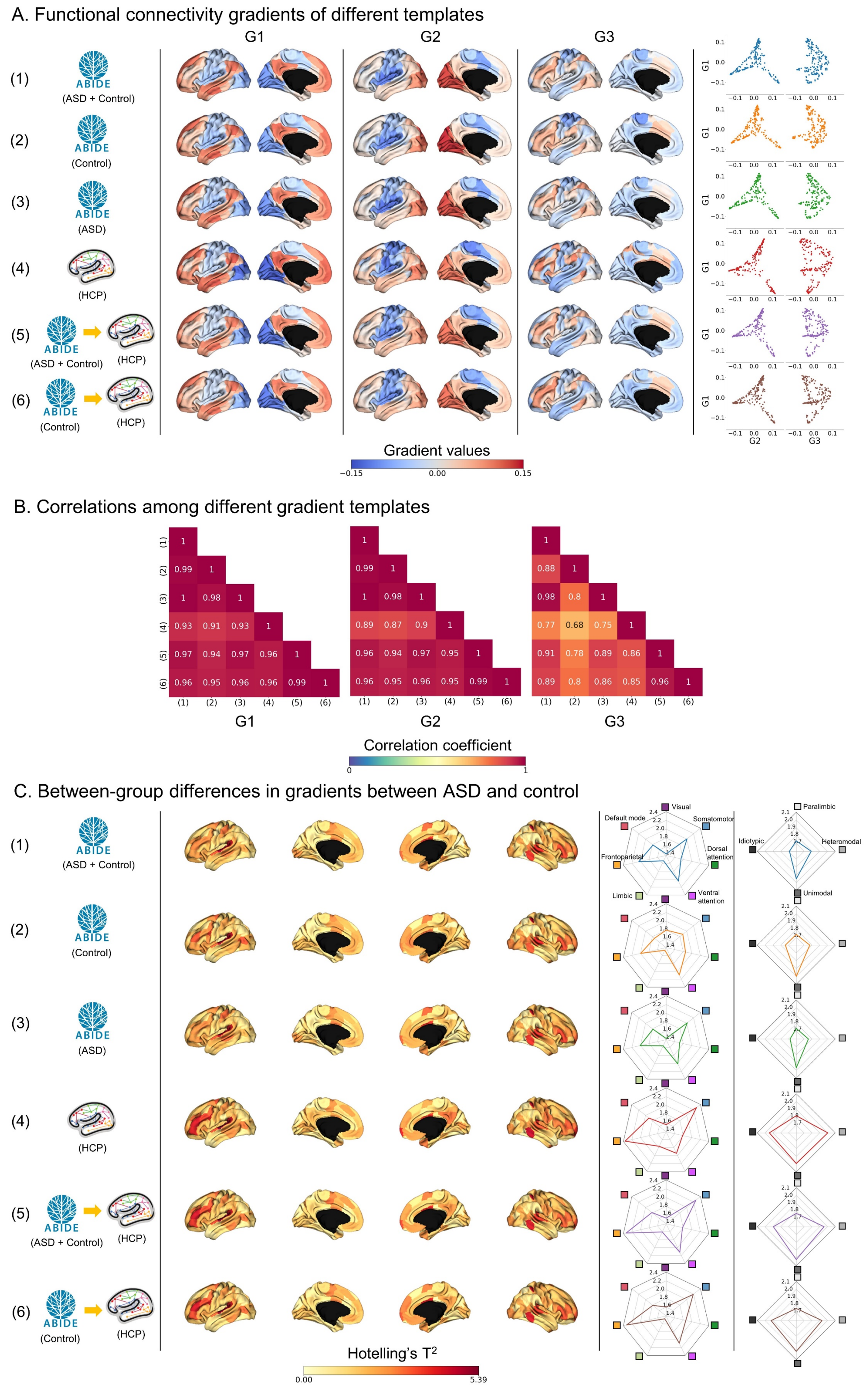

Supplementary Fig. 21 | Functional gradients of different templates and between-group differences in NYU site. (A) We generated three functional connectivity gradients (G1, G2, and G3) of six different template strategies and plotted them on brain surfaces. The scatter plots display the distribution of gradient pairs. **(B)** We reported heatmaps of correlation coefficients between different template pairs. **(C)** Hotelling's T^2 statistics of the whole brain are plotted on brain surfaces. We stratified the effects according to seven functional communities and four cortical hierarchical levels using radar plots. Abbreviations: ASD, autism spectrum disorder; HCP, Human Connectome Project; ABIDE, Autism Brain Imaging Data Exchange Initiative.

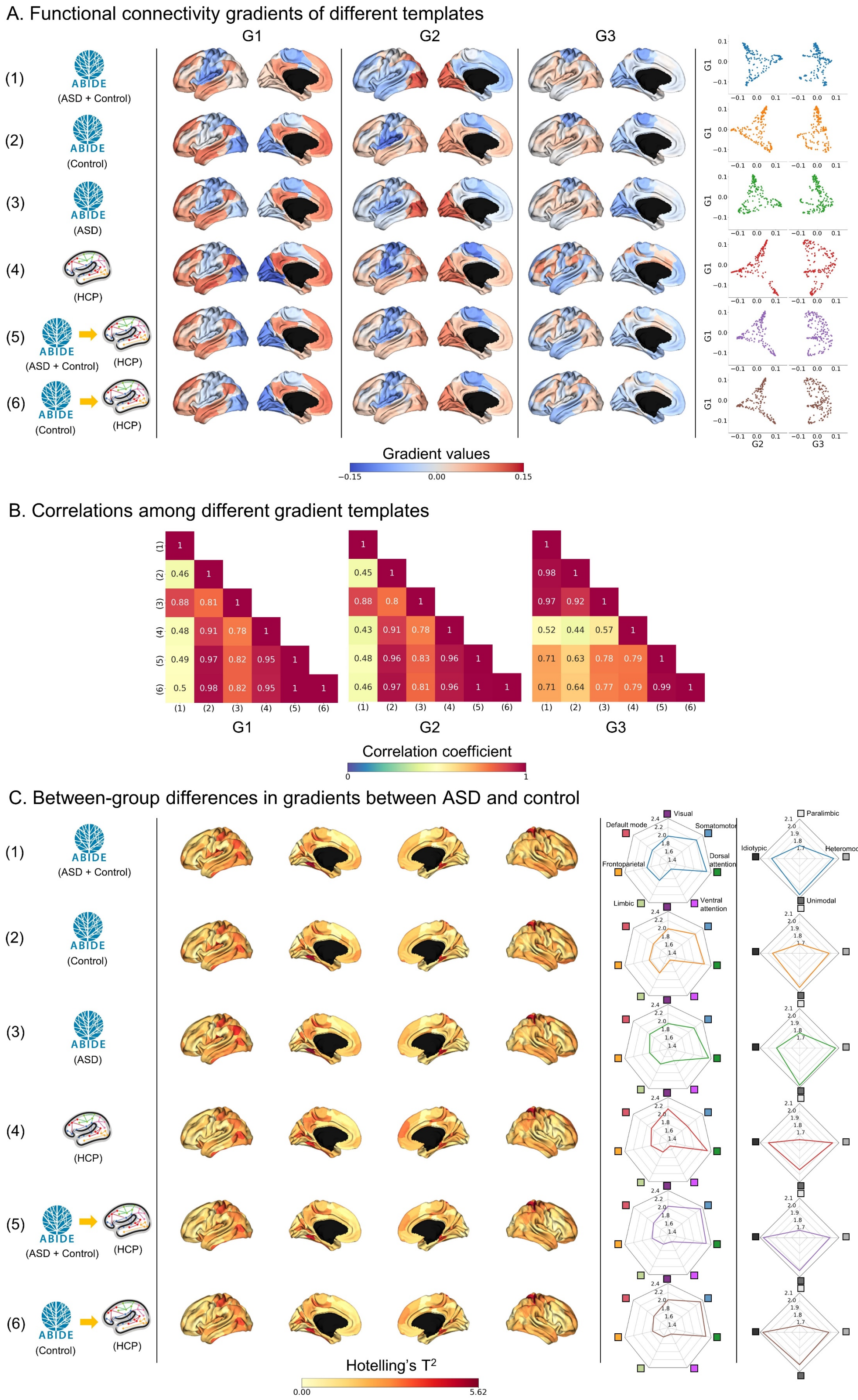

Supplementary Fig. 22 | Functional gradients of different templates and between-group differences in TCD site. (A) We generated three functional connectivity gradients (G1, G2, and G3) of six different template strategies and plotted them on brain surfaces. The scatter plots display the distribution of gradient pairs. **(B)** We reported heatmaps of correlation coefficients between different template pairs. **(C)** Hotelling's T^2 statistics of the whole brain are plotted on brain surfaces. We stratified the effects according to seven functional communities and four cortical hierarchical levels using radar plots. Abbreviations: ASD, autism spectrum disorder; HCP, Human Connectome Project; ABIDE, Autism Brain Imaging Data Exchange Initiative.

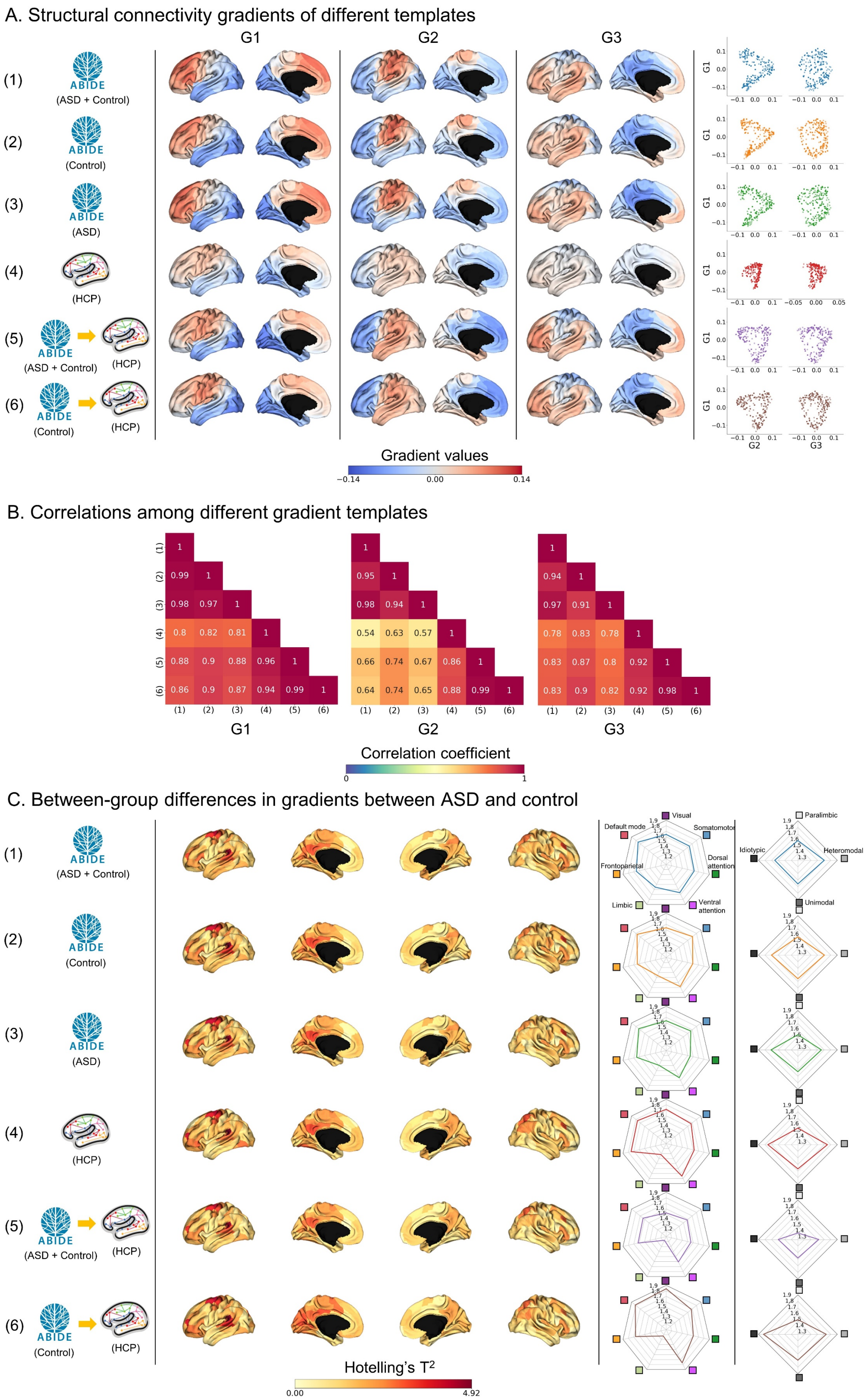

Supplementary Fig. 23 | Structural gradients of different templates and between-group differences in IP site. (A) We generated three structural connectivity gradients (G1, G2, and G3) of six different template strategies and plotted them on brain surfaces. The scatter plots display the distribution of gradient pairs. **(B)** We reported heatmaps of correlation coefficients between different template pairs. **(C)** Hotelling's T^2 statistics of the whole brain are plotted on brain surfaces. We stratified the effects according to seven functional communities and four cortical hierarchical levels using radar plots. Abbreviations: ASD, autism spectrum disorder; HCP, Human Connectome Project; ABIDE, Autism Brain Imaging Data Exchange Initiative.

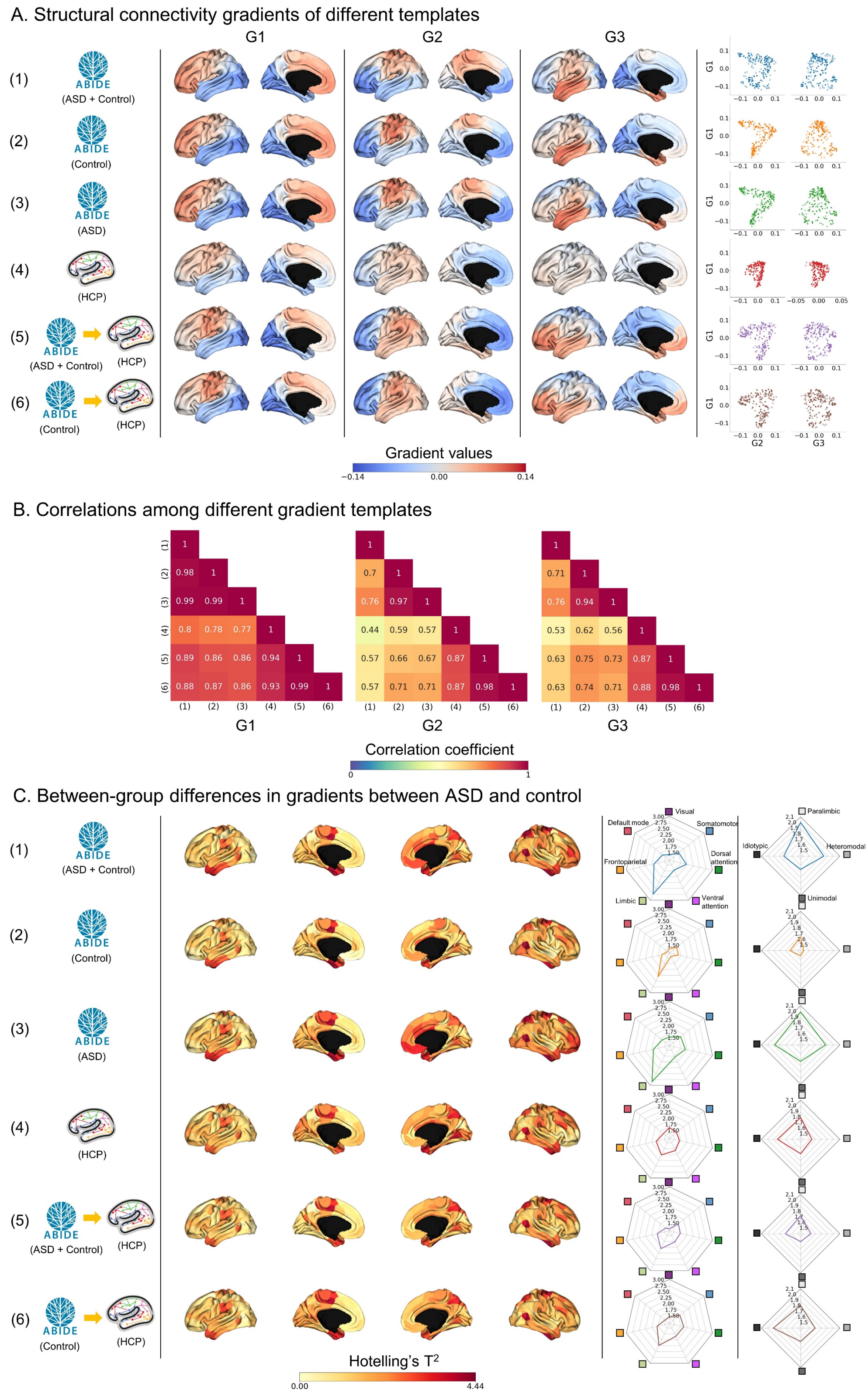

Supplementary Fig. 24 | Structural gradients of different templates and between-group differences in NYU site. (A) We generated three structural connectivity gradients (G1, G2, and G3) of six different template strategies and plotted them on brain surfaces. The scatter plots display the distribution of gradient pairs. **(B)** We reported heatmaps of correlation coefficients between different template pairs. **(C)** Hotelling's T^2 statistics of the whole brain are plotted on brain surfaces. We stratified the effects according to seven functional communities and four cortical hierarchical levels using radar plots. Abbreviations: ASD, autism spectrum disorder; HCP, Human Connectome Project; ABIDE, Autism Brain Imaging Data Exchange Initiative.

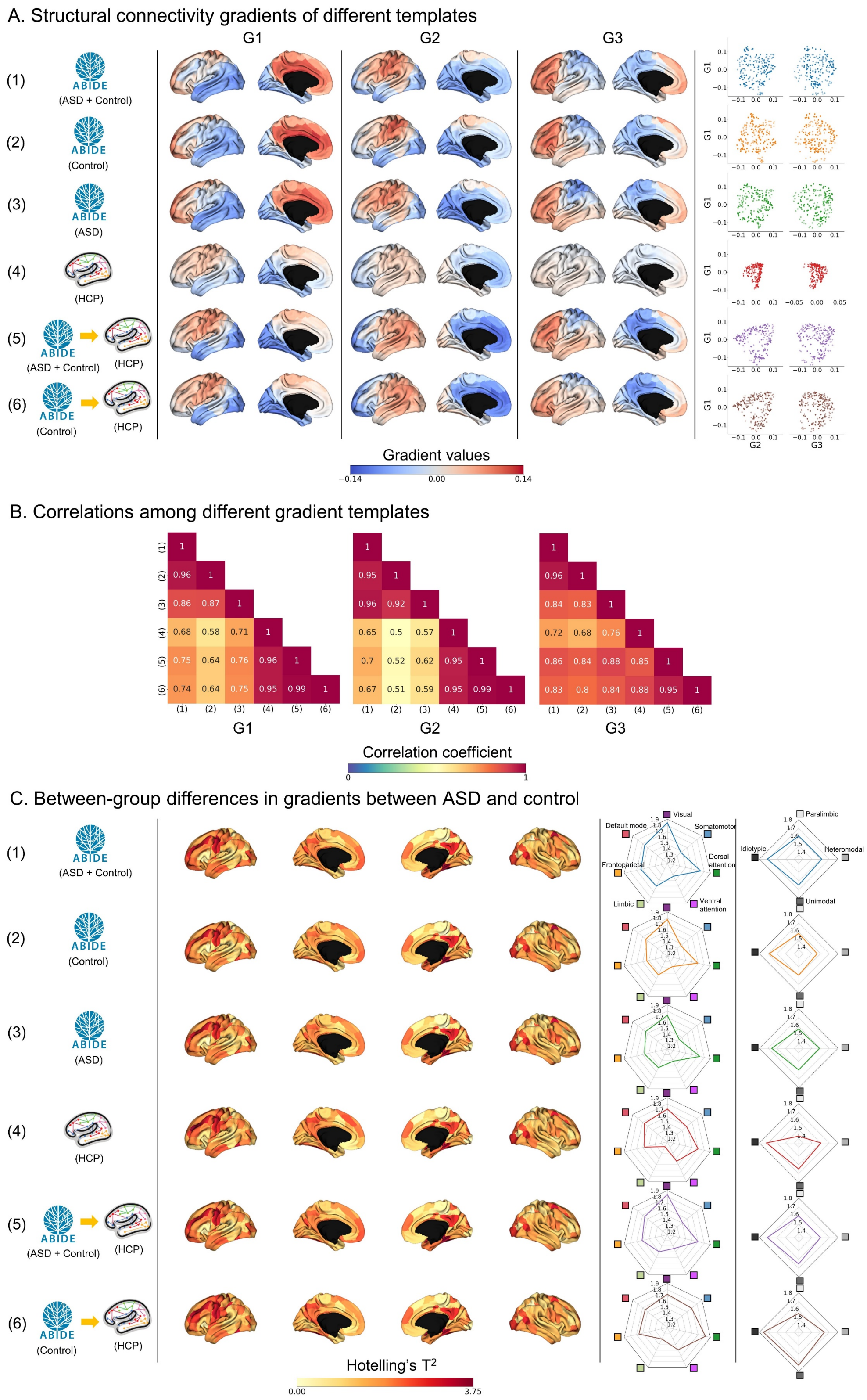

Supplementary Fig. 25 | Structural gradients of different templates and between-group differences in TCD site. (A) We generated three structural connectivity gradients (G1, G2, and G3) of six different template strategies and plotted them on brain surfaces. The scatter plots display the distribution of gradient pairs. **(B)** We reported heatmaps of correlation coefficients between different template pairs. **(C)** Hotelling's T^2 statistics of the whole brain are plotted on brain surfaces. We stratified the effects according to seven functional communities and four cortical hierarchical levels using radar plots. Abbreviations: ASD, autism spectrum disorder; HCP, Human Connectome Project; ABIDE, Autism Brain Imaging Data Exchange Initiative.

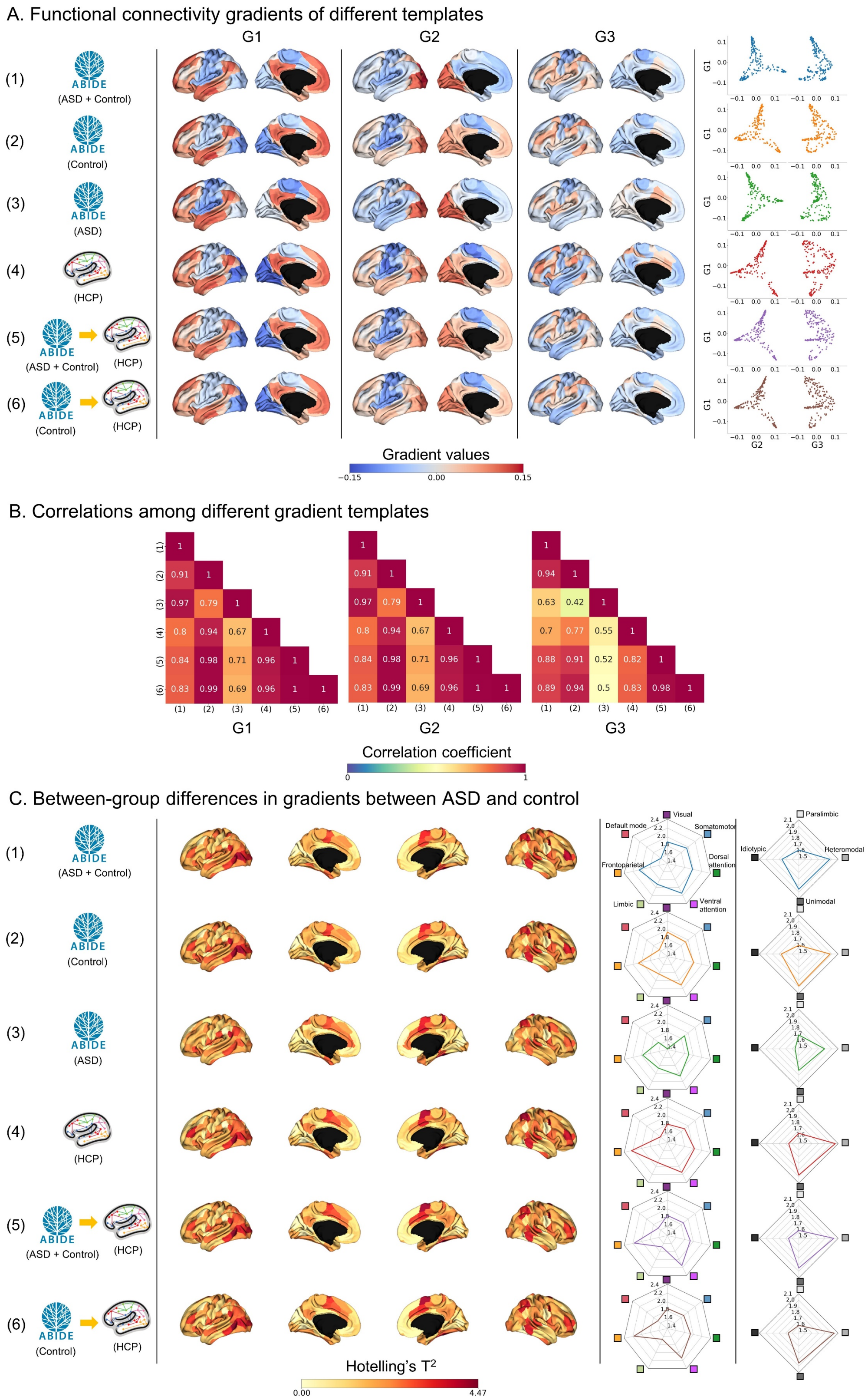

Supplementary Fig. 26 | Functional gradients of different templates and between-group differences using only adults. **(A)** We generated three functional connectivity gradients (G1, G2, and G3) of six different template strategies and plotted them on brain surfaces. The scatter plots display the distribution of gradient pairs. **(B)** We reported heatmaps of correlation coefficients between different template pairs. **(C)** Hotelling's T^2 statistics of the whole brain are plotted on brain surfaces. We stratified the effects according to seven functional communities and four cortical hierarchical levels using radar plots. Abbreviations: ASD, autism spectrum disorder; HCP, Human Connectome Project; ABIDE, Autism Brain Imaging Data Exchange Initiative.

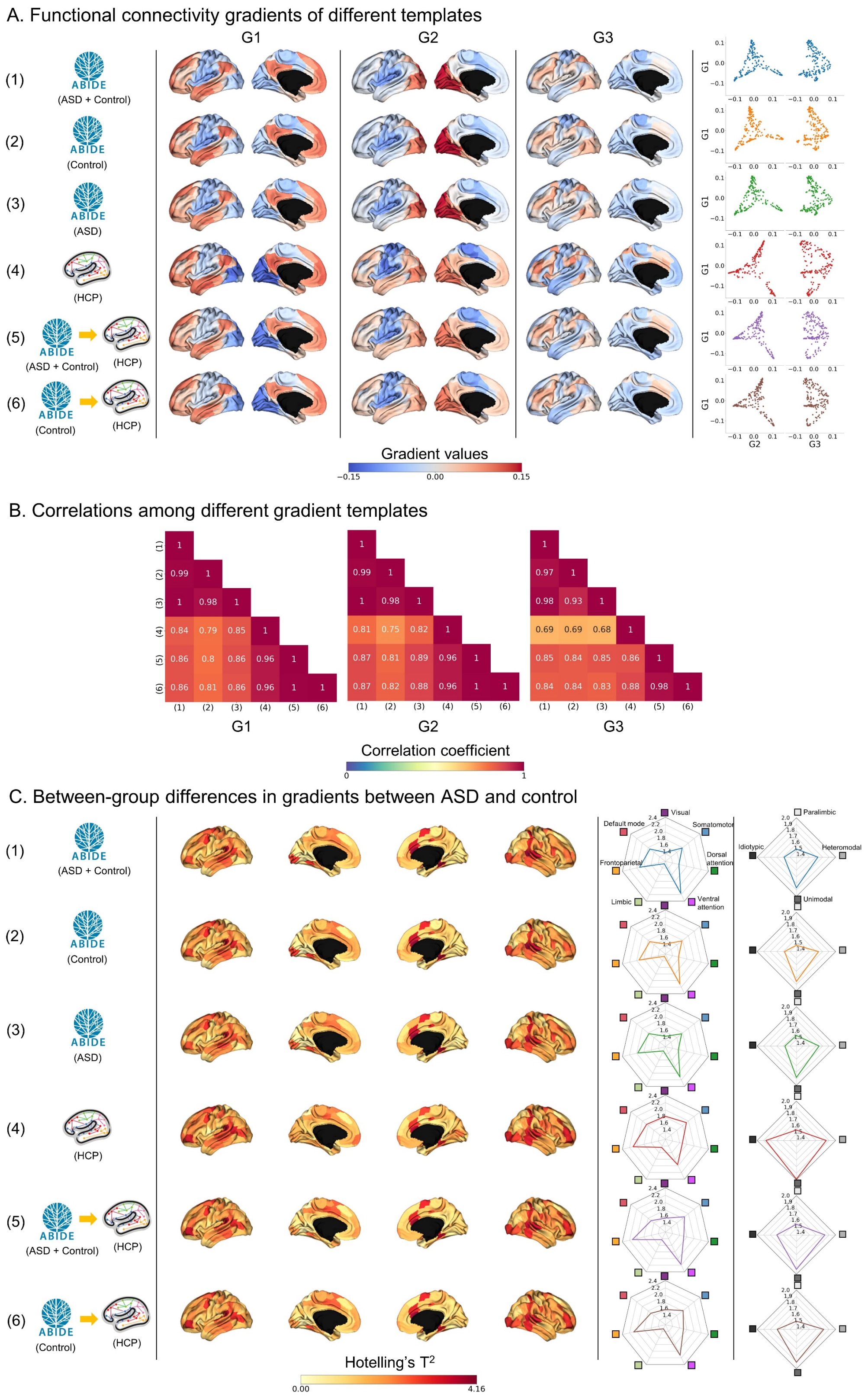

Supplementary Fig. 27 | Functional gradients of different templates and between-group differences using only children. (A) We generated three functional connectivity gradients (G1, G2, and G3) of six different template strategies and plotted them on brain surfaces. The scatter plots display the distribution of gradient pairs. **(B)** We reported heatmaps of correlation coefficients between different template pairs. **(C)** Hotelling's T^2 statistics of the whole brain are plotted on brain surfaces. We stratified the effects according to seven functional communities and four cortical hierarchical levels using radar plots. Abbreviations: ASD, autism spectrum disorder; HCP, Human Connectome Project; ABIDE, Autism Brain Imaging Data Exchange Initiative.

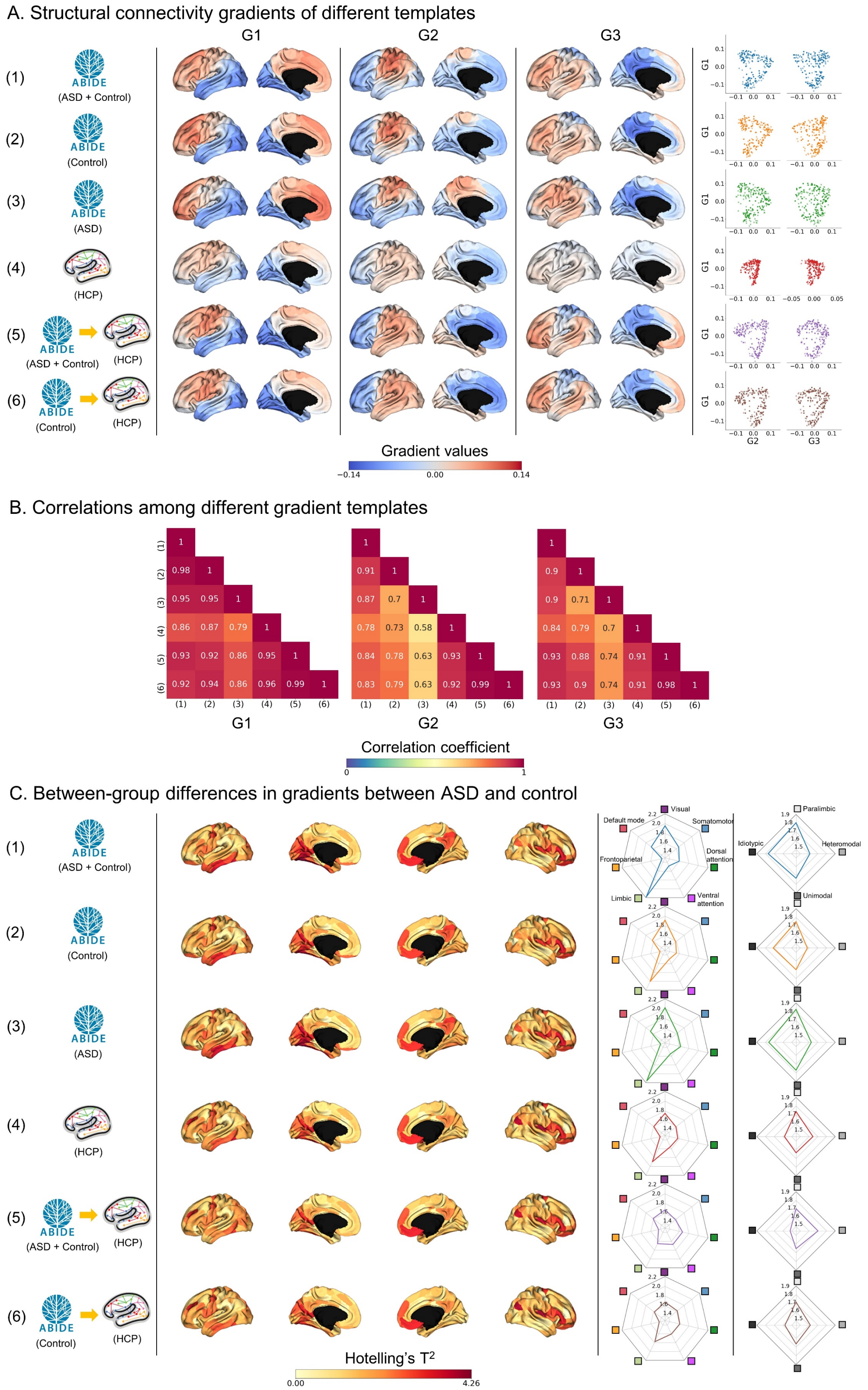

Supplementary Fig. 28 | Structural gradients of different templates and between-group differences using only adults. **(A)** We generated three structural connectivity gradients (G1, G2, and G3) of six different template strategies and plotted them on brain surfaces. The scatter plots display the distribution of gradient pairs. **(B)** We reported heatmaps of correlation coefficients between different template pairs. **(C)** Hotelling's T^2 statistics of the whole brain are plotted on brain surfaces. We stratified the effects according to seven functional communities and four cortical hierarchical levels using radar plots. Abbreviations: ASD, autism spectrum disorder; HCP, Human Connectome Project; ABIDE, Autism Brain Imaging Data Exchange Initiative.

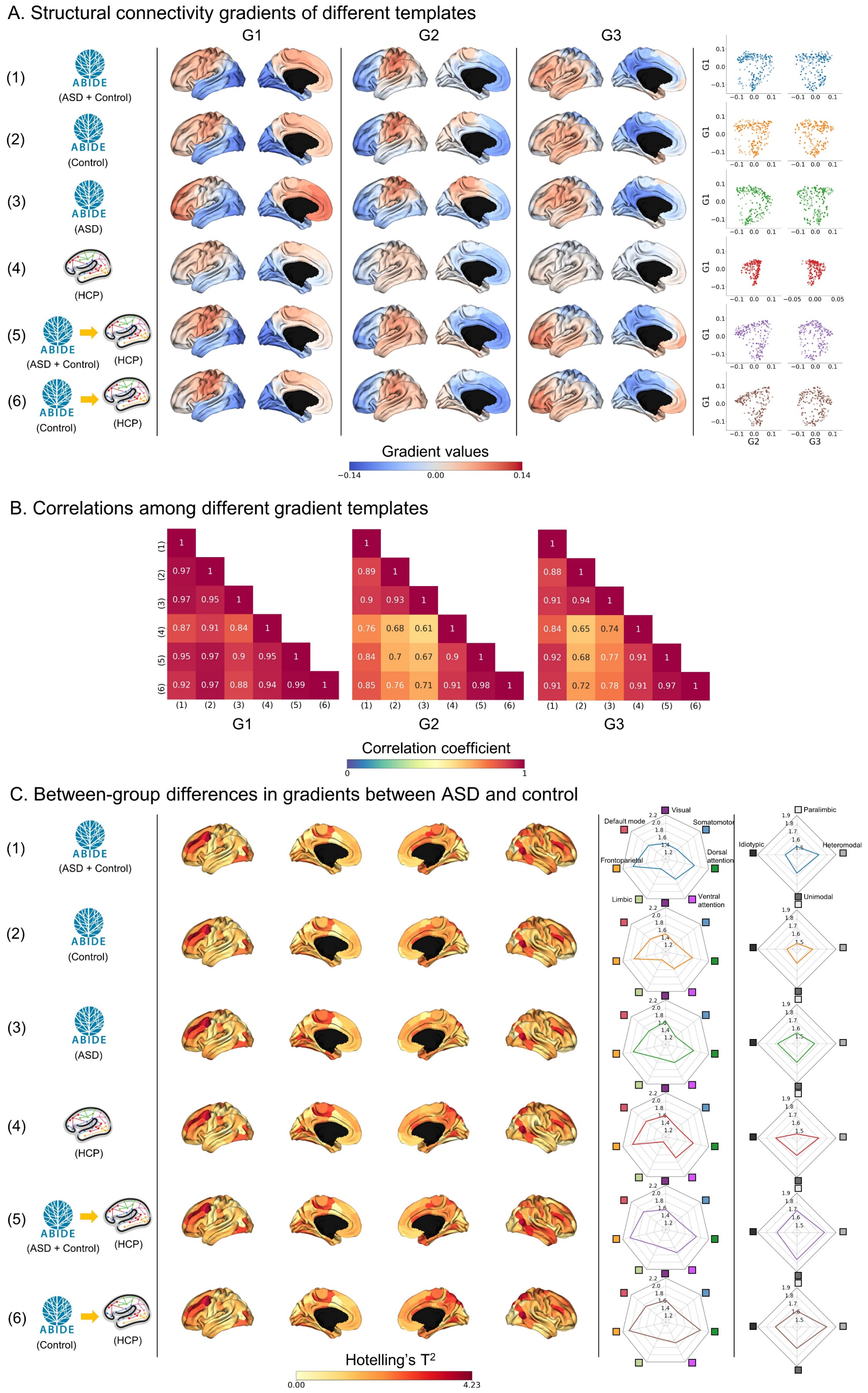

Supplementary Fig. 29 | Structural gradients of different templates and between-group differences using only children. (A) We generated three structural connectivity gradients (G1, G2, and G3) of six different template strategies and plotted them on brain surfaces. The scatter plots display the distribution of gradient pairs. **(B)** We reported heatmaps of correlation coefficients between different template pairs. **(C)** Hotelling's T^2 statistics of the whole brain are plotted on brain surfaces. We stratified the effects according to seven functional communities and four cortical hierarchical levels using radar plots. Abbreviations: ASD, autism spectrum disorder; HCP, Human Connectome Project; ABIDE, Autism Brain Imaging Data Exchange Initiative.

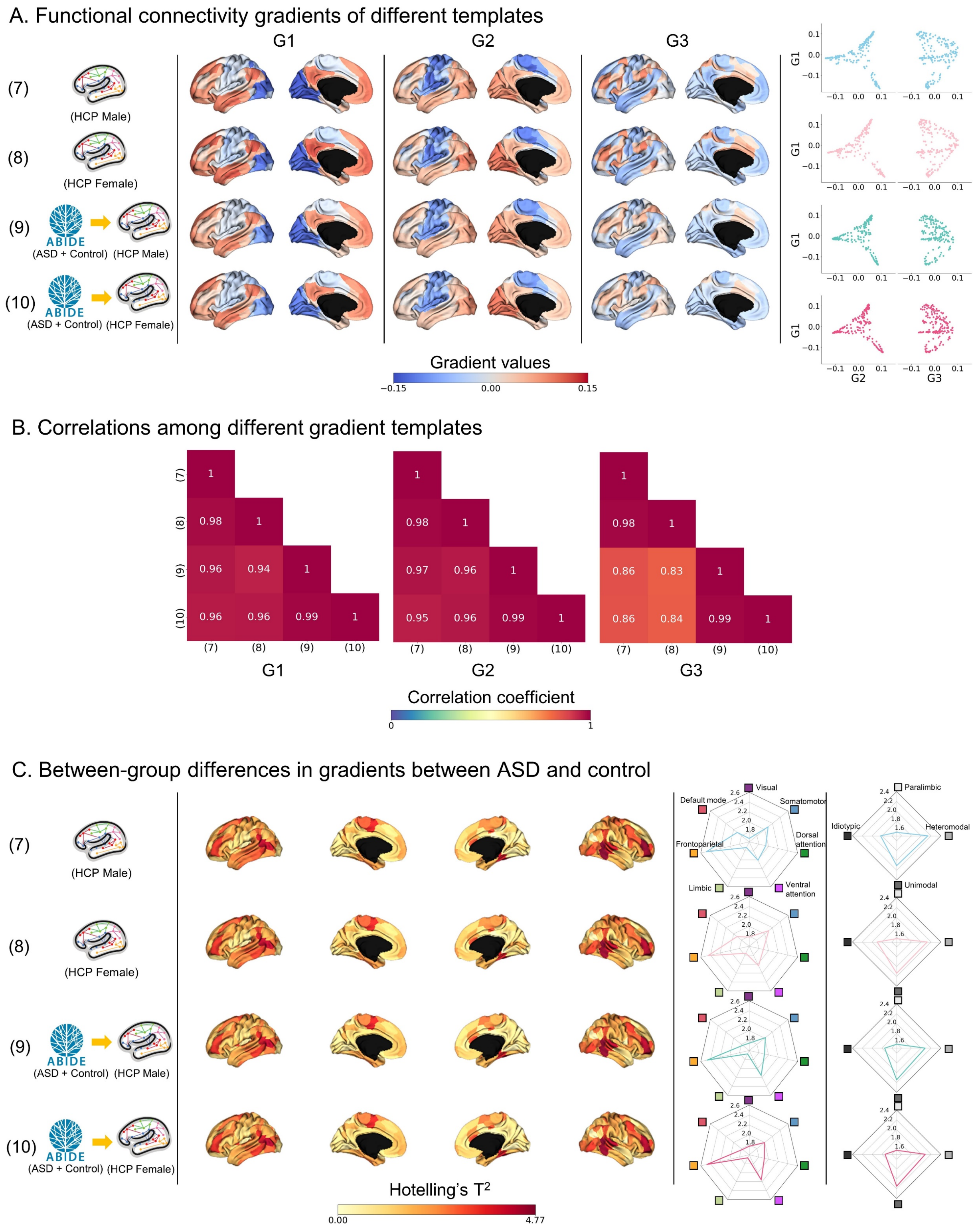

Supplementary Fig. 30 | Functional connectivity gradients of sex-specific templates and between-group differences. **(A)** We generated three functional connectivity gradients (G1, G2, and G3) of sex-specific templates and plotted them on brain surfaces. The scatter plots display the distribution of gradient pairs. **(B)** We reported heatmaps of correlation coefficients between different template pairs. **(C)** Hotelling's T^2 statistics of the whole brain are plotted on brain surfaces. We stratified the effects according to seven functional communities and four cortical hierarchical levels using radar plots. Abbreviations: ASD, autism spectrum disorder; HCP, Human Connectome Project; ABIDE, Autism Brain Imaging Data Exchange Initiative.

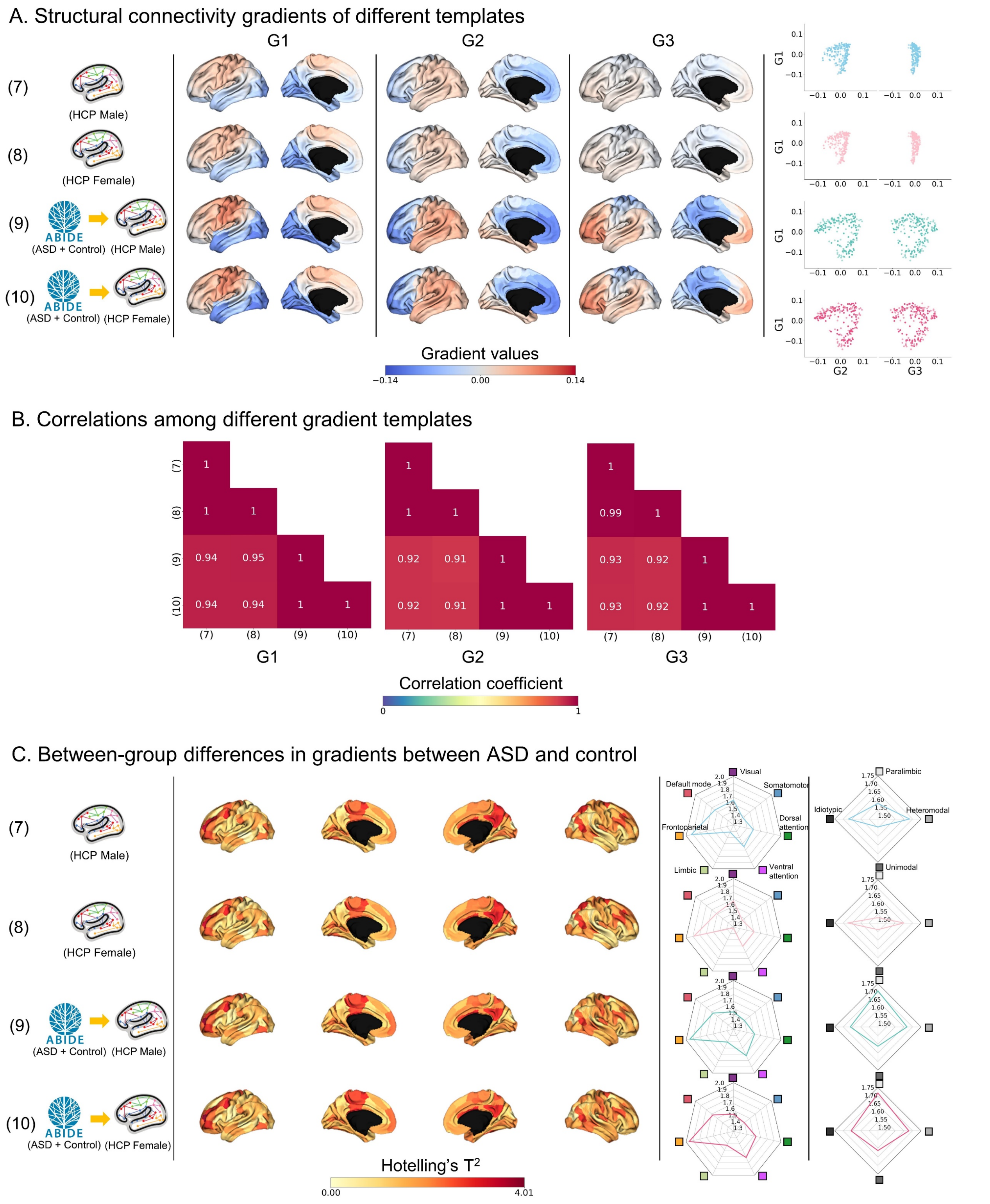

Supplementary Fig. 31 | Structural connectivity gradients of sex-specific templates and between-group differences. **(A)** We generated three structural connectivity gradients (G1, G2, and G3) of sex-specific templates and plotted them on brain surfaces. The scatter plots display the distribution of gradient pairs. **(B)** We reported heatmaps of correlation coefficients between different template pairs. **(C)** Hotelling's T^2 statistics of the whole brain are plotted on brain surfaces. We stratified the effects according to seven functional communities and four cortical hierarchical levels using radar plots. Abbreviations: ASD, autism spectrum disorder; HCP, Human Connectome Project; ABIDE, Autism Brain Imaging Data Exchange Initiative.
